# Probing the pH-dependency of DC-SIGN/R multivalent lectin–glycan interactions using polyvalent glycan-gold nanoparticles†

**DOI:** 10.1039/d3na01013a

**Published:** 2024-03-11

**Authors:** Rahman Basaran, Xinyu Ning, Darshita Budhadev, Nicole Hondow, Yuan Guo, Dejian Zhou

**Affiliations:** a School of Chemistry, Astbury Centre for Structural Molecular Biology, University of Leeds Leeds LS2 9JT UK d.zhou@leeds.ac.uk; b School of Chemical and Process Engineering, University of Leeds Leeds LS2 9JT UK; c School of Food Science and Nutrition, Astbury Centre for Structural Molecular Biology, University of Leeds Leeds LS2 9JT UK y.guo@leeds.ac.uk

## Abstract

The dendritic cell tetrameric lectin, DC-SIGN, and its closely related endothelial cell lectin, DC-SIGNR (collectively abbreviated as DC-SIGN/R) play a key role in the binding and transmission of deadly viruses, including Ebola, HIV, HCV, and SARS-CoV-2. Their virus binding/release processes involve a gradually acidifying environment following the natural intracellular trafficking pathways. Therefore, understanding DC-SIGN/R's pH-dependent binding properties with glycan ligands is of great importance. We have recently developed densely glycosylated gold nanoparticles (glycan-GNPs) as a powerful new tool for probing DC-SIGN/R multivalent lectin–glycan interaction (MLGI) mechanisms. They can provide not only quantitative MLGI affinities but also important structural information, such as binding site orientation and binding modes. Herein, we further employ the glycan-GNP probes to investigate the pH dependency of DC-SIGN/R MLGI properties. We find that DC-SIGN/R MLGIs exhibit distinct pH dependence over the normal physiological (7.4) to lysosomal (∼4.6) pH range. DC-SIGN binds glycan-GNPs strongly and stably from pH 7.4 to ∼5.8, but the binding is weakened significantly as pH decreases to ≤5.4 and may be fully dissociated at pH 4.6. This behaviour is fully consistent with DC-SIGN's role as an endocytic recycling receptor. In contrast, DC-SIGNR's affinity with glycan-GNPs is enhanced with the decreasing pH from 7.4 to 5.4, peaking at pH 5.4, and then reduced as pH is further lowered. Interestingly, both DC-SIGN/R binding with glycan-GNPs are found to be partially reversible in a pH-dependent manner.

## Abstract 2

DC-SIGN displays distinct pH-dependent binding with GNP-DiMan, strong tetravalent binding to single GNP-DiMan at pH 7.4, weak crosslinking at pH 5.4, and no apparent binding at pH 4.6, consistent with its biological role as an endocytic recycling receptor.

## Graphical abstract

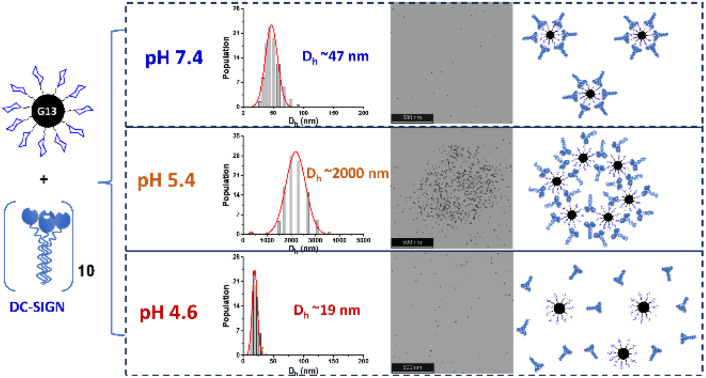

## Introduction

Multivalent lectin–glycan interactions (MLGIs) are widely employed in biology and play a critical role in modulating many essential biological functions, including the recognition and signalling of invading pathogens and modulation of host cell immune responses. Most pathogens target host cells by forming multivalent interactions with cell surface lectins *via* their surface specific glycans (or *vice versa*) to gain entry to host cells to initiate infection.^1,2^ As monovalent interactions between lectins and carbohydrates are characteristically weak, with dissociation constants (*K*_d_s) typically in the mM range,^3^ they are too weak to produce a biological response. To compensate for this, most lectins form oligomeric structures which cluster multiple carbohydrate recognition domains (CRDs) together to form multivalent binding with glycans to enhance affinity to make bindings biologically functional.^4,5^ The infection processes of many viruses, including HIV, Ebola, West Nile,^6–10^ and more recently, SARS-CoV-2, are mainly initiated or facilitated (in the case of SARS-CoV-2)^11^ by binding of viral surface glycans to host cell multimeric lectins (or *vice versa*). Therefore, elucidating the mechanisms which glycoconjugates form strong and specific MLGIs with multimeric lectins is of great importance and significance, allowing us to design effective glycoconjugates to potently block specific MLGIs, thereby preventing viral infections.^4,12–17^ Compared to other approaches, this antiviral strategy has a unique advantage because it can effectively prevent viral mutation and acquire resistance.^14,18^ While free glycans can be directly employed for this purpose, they are unlikely to be effective because of their weak monovalent binding affinity with target lectins. By displaying multiple glycans onto suitable nanoscale scaffolds, the resulting polyvalent glycoconjugates can bind multivalently with multimeric lectins, resulting in greatly enhanced MLGI affinity, up to 5–6 orders of magnitude, over the corresponding monovalent affinity.^7,13–17,19,20^ In this regard, nanomaterials are robust scaffolds for displaying multivalent glycans for potent lectin targeting. In particular, gold nanoparticles, GNPs, are well-suited for constructing polyvalent glycoconjugates, owing to their advantageous properties, such as low-/nontoxicity,^21^ widely available size and shape range, and robust gold–thiol surface chemistry for easy tuning of the glycan density and valency.^15,17,20,22^ Notably, their large surface-area-to-volume ratio is also advantageous in forming stable, well-presented three-dimensional displays of target glycans.^12,17,20^ Furthermore, glycan-functionalised GNPs (glycan-GNPs) have excellent colloidal stability, high biocompatibility and resistance against non-specific interactions; these make them well-suited for a wide range of biological and biomedical applications.^9,17,20,23^

Meanwhile, pH is a vitally important environmental stimulus for many biological processes and functions. It also plays a key role in viral infections. Viruses often attach to host cells by binding their surface specific glycans to host cell lectin receptors (or *vice versa*) to gain cell entry and infection. In particular, the dendritic cell surface tetrameric lectin, DC-SIGN,^9^ and its closely related endothelial cell surface lectin, DC-SIGNR,^10^ (collectively abbreviated as DC-SIGN/R hereafter), play a key role in binding the HIV^24^ and Ebola virus (EBOV)^25^ to augment viral entry and infection. After binding, viruses are internalised into host cells, mainly into endosomes, and then subsequently trafficked to lysosomes following the natural endocytotic and trafficking pathways, during which, the bound lectin-virus complexes are exposed to a gradually acidified environment in such intracellular compartments, which plays a crucial role in the infectivity of viruses.^26^ The weakly acidic environment of early (pH ∼ 6) or late (pH ∼ 5) endosomes^27^ generally leads to the dissociation of lectin-virus complexes, which is important for virus endosomal escape to retain infectivity. In case no dissociation happens in endosomes, the complexes may be trafficked to lysosomes (pH ∼ 4.6) for degradation; this often results in the loss of viral infectivity.^28,29^ Therefore, understanding the pH-dependent glycan binding and releasing properties of DC-SIGN/R is of great interest and importance to biology, although the resulting processes are still not fully understood.

Interestingly, despite close similarities in the overall tetrameric architecture and identical monovalent CRD–mannose binding motifs,^28,30^ DC-SIGN/R actually display distinct virus binding and trans-infection properties. For example, DC-SIGN is more effective in transmitting the HIV infection than DC-SIGNR,^31^ while only DC-SIGNR, but not DC-SIGN, can transmit the West Nile virus infection.^32^ The structural mechanisms underlying such differences remain not fully understood. By developing a new glycan-nanoparticle based multifunctional probe, we have discovered that DC-SIGN/R clearly exhibit distinct modes and affinities in binding to glycan-nanoparticles, due to subtle differences in their binding site orientation.^6,7^ All four binding sites in DC-SIGN point upward in the same direction, but those in DC-SIGNR are split into two pairs and point side-ways. As a result, DC-SIGN binds tetravalently to a single glycan-nanoparticle, whereas DC-SIGNR binds bisdivalently with two different glycan-nanoparticles, resulting in the former binding being significantly stronger than the latter (by ∼20–200 fold).^7,20^ Despite success, how environmental pH affects the solution MLGI properties between DC-SIGN/R and glycan-nanoparticles remains to be elucidated. Moreover, our earlier glycan-nanoparticle probes were built upon small nanoparticle scaffolds of 4–5 nm in diameters,^7,20^ which may not be optimal in terms of potency and specificity against target lectins for biomedical applications. Indeed, the scaffold size of multivalent glycofullerene-nanoparticles has been shown to strongly affect their anti-adhesive properties against several bacterial and viral pathogens, with 20 nm being found to be optimal.^17^ In this paper, we have prepared densely glycosylated gold nanoparticles (glycan-GNPs) of two different sizes (*e.g.*, ∼13 and ∼27 nm in diameter) and systematically investigated their pH-dependent MLGI properties with DC-SIGN/R over a pH range of 4.6 to 7.4, mimicking the pH range experienced by viruses during the natural cellular uptake and trafficking pathways through endocytosis. We have studied their relative MLGI affinities as a function of pH by exploiting GNP's strong fluorescence quenching properties^33^ and further monitored the hydrodynamic diameters (*D*_h_s) of the resulting lectin–glycan-GNP complexes, revealing that the MLGIs of DC-SIGN/R display distinct pH dependency. We have further investigated the reversibility of DC-SIGN/R–glycan-GNP binding by cycling pH between 4.6 and 7.4, showing that the process is reversible. Our results thus provide a useful insight into the pH-dependent glycan binding and release properties of these important lectin viral receptors.

## Results and discussion

### GNP synthesis and characterisation

Spherical GNPs with average diameters of ∼13 nm and ∼27 nm were employed to construct glycan-GNPs. As most virus surface trimeric glycoprotein spikes are ∼13 nm in size, for example, the HIV surface trimeric gp160 spike,^34^ the 13 nm GNP (abbreviated as G13) was used to mimic virus spikes. Furthermore, a 27 nm GNP (G27) was also employed to investigate how GNP scaffold size may impact glycan-GNP's pH-dependent binding with DC-SIGN/R. G13 was synthesised using the standard citrate reduction method as described previously. G27 was synthesised by citrate reduction with the addition of a small amount of NaOH by following a literature protocol.^35,36^ Their detailed synthesis procedures are given in the Experimental section. The average GNP core diameters were determined by TEM to be ∼13 nm for G13 and ∼27 nm for G27, respectively. Both G13 and G27 solutions gave a single plasmonic absorption peak at ∼520 and ∼522 nm, respectively, consistent with those expected for isolated single GNPs. They both displayed a single volume size population with a mean hydrodynamic diameter (*D*_h_) of ∼16 nm (polydispersity index, PDI, = 0.22) for G13 and a *D*_h_ of ∼29 nm (PDI = 0.21) for G27, confirming that they were uniform, aggregation-free GNPs (see ESI, Fig. S1 and S2† for their UV-vis spectra, TEM images and DLS histograms). The concentrations of G13 and G27 were obtained using the Beer–Lambert law from their SPR peak absorbance at 520 or 522 nm using a molar absorption extinction coefficient of 2.32 × 10^8^ or 2.39 × 10^9^ M^−1^ cm^−1^ for G13 or G27, respectively.^20,36^

### Ligand design and synthesis

**Fig. 1:**
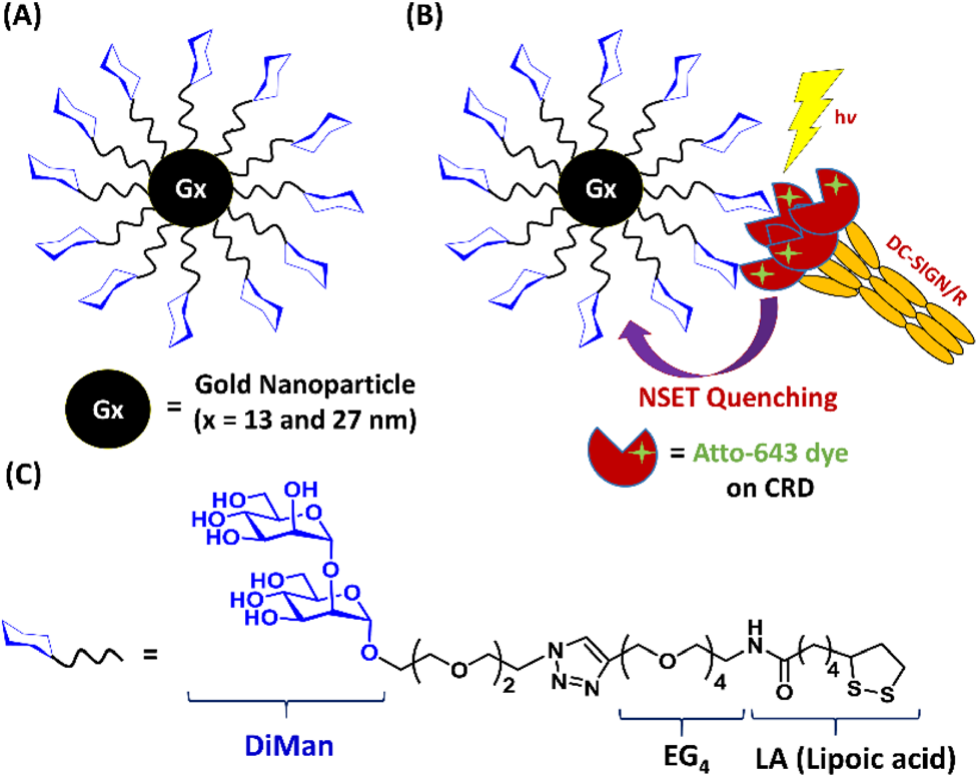
(A) Schematic structure of G*x*-DiMan; (B) our approach to probe DC-SIGN/R binding with G*x*-DiMan *via* GNP fluorescence quenching: upon binding, the excited energies of fluorophore labels on DC-SIGN/R are efficiently transferred to the proximal G*x*-DiMan *via* the NSET mechanism, leading to a greatly reduced fluorescence signal. (C) Chemical structure of the LA–EG_4_–DiMan ligand.

A lipoic acid-tetra(ethylene glycol)-α-1-manno-α-1,2-biose (abbreviated as LA–EG_4_–DiMan)-based multifunctional glycan ligand was synthesised *via* our established procedures.^20^ We have chosen α-1-manno-α-1,2-biose (DiMan) as the target glycan because it is relatively easy to synthesise. Moreover, a polyvalent display of DiMan on GNPs has been shown to give stronger MLGI affinities with DC-SIGN than some more complex (oligo)mannosides.^37^ The LA–EG_4_–DiMan ligand was designed to contain three unique functional domains: a lipoic acid (LA) group to provide strong chelative binding to the GNP surface by forming 2 strong Au–S bonds;^20,38^ a flexible, hydrophilic tetra(ethylene glycol) (EG_4_) linker to promote high stability, water solubility, and excellent resistance against non-specific interactions and adsorptions (ensuring all results measured are due to specific interactions only);^20,39^ and a terminal α-1-manno-α-1,2-biose group to afford specific binding with DC-SIGN/R (Fig. 1). The LA–EG_4_–DiMan ligand was synthesised by Cu-catalysed click chemistry between a LA–EG_4_–acetylene linker and N_3_–EG_2_–DiMan and purified by using a P2 biogel column as reported previously.^20,40^ Details of the ligand synthesis and purification procedures and its spectroscopic characterisation are provided in the ESI, Fig. S3†.

### GNP-glycan preparation and characterisation

LA–EG_4_–DiMan ligand capped GNPs (G*x*-DiMan, *x* = 13 or 27) were prepared by treating the citrate-stabilised GNPs with LA–EG_4_–DiMan in an aqueous solution.^20^ Briefly, GNPs were incubated with the LA–EG_4_–DiMan ligand at a fixed GNP : ligand molar ratio of 1 : 3000 for G13 or 1 : 10 000 for G27. The resulting solutions were stirred at room temperature (RT) in darkness (wrapped with foil) overnight to complete the GNP glycosylation *via* self-assembly. Any unbound free ligands were removed by centrifugation followed by washing with pure water. The successful preparation of G*x*-DiMan was supported by greatly improved resistance against salt induced aggregation (citrate stabilised GNPs readily aggregate and produce colour changes upon addition of NaCl salt which can effectively screen their electrostatic repulsions).^41^ Moreover, an increase in *D*_h_ by a few nm was also observed for both G13 (from ∼16 to ∼22 nm) and G27 (from ∼29 to ∼32 nm) after glycosylation. This result was consistent with what was expected for G13 or G27 coated with a self-assembled monolayer of LA–EG_4_–DiMan molecules. The G*x*-DiMan solutions were found to be highly stable, and no changes in physical appearance or precipitation were observed after storage at 4 °C for more than one year. Moreover, the UV-vis spectra of G*x*-DiMan overlaid well with those of the parent citrate stabilised GNPs with no significant red-shift and broadening of the SPR peaks (ESI, Fig. S4†), suggesting that no aggregation had taken place. The average number of glycan ligands bound on each GNP was estimated to be ∼2200 ± 170 and ∼6290 ± 440 for G13-DiMan and G27-DiMan, respectively, by measuring the ligand amount difference between that added and that remained unbound in the supernatant after GNP conjugation, using the phenol sulphuric acid carbohydrate quantitation method described previously. The average inter-glycan distances (*d*) were estimated to be ∼0.93 and ∼0.80 nm for G13-DiMan and G27-DiMan, respectively, based on the G*x*-DiMan *D*_h_ values and glycan valencies *via* the methods described previously (see ESI, Table S1†).^20^ These values match well to the major inter-glycan sequon spaces (*e.g.*, 0.7–1.3 nm) found on the HIV surface densely glycosylated gp160 trimers, which are responsible for the HIV–DC-SIGN binding to initiate infection. Thus, G*x*-DiMan may serve as a good mimic for HIV gp160 to probe its multivalent binding interactions with DC-SIGN/R.

### DC-SIGN/R production and labelling

The soluble extracellular segments of DC-SIGN/R (denoted as DC-SIGN/R hereafter) have been shown to faithfully replicate the tetrameric structures and glycan binding properties of the full length DC-SIGN/R;^6,7^ therefore they were employed to investigate the pH dependency of DC-SIGN/R binding interactions with G*x*-DiMan. To facilitate sensitive fluorescence readout, the residue Q274 in DC-SIGN or R287 in DC-SIGNR was mutated to a cysteine residue for site-specific dye labelling.^6,7,30^ These residues are located close to, but outside of, the glycan binding pocket on the CRD; hence dye labelling does not affect their glycan binding properties, as confirmed previously.^6,7^ The constructed DC-SIGN Q274C and DC-SIGNR R287C were expressed in *E*. *coli*, purified by affinity column chromatography, and then site-specifically labelled with maleimide modified Atto-643 as described previously.^7,20^ We have chosen Atto-643 as the fluorescent label here because of its high fluorescence quantum yield, excellent photo-stability, and strong hydrophilicity (minimising the possibility of forming aggregates and/or inducing non-specific interactions in aqueous media). Moreover, its fluorescence is stable over a pH range of 2 to 11, making it a robust fluorescence probe under a wide range of biological conditions.^42^ Furthermore, its absorption and emission occur in the red region of the visible spectrum where GNPs have minimal absorption, which can greatly reduce the possible contribution of fluorescence quenching from the GNP's inner filter effect. The dye labelling efficiency was estimated to be ∼82% and ∼90% per monomer for DC-SIGN and DC-SIGNR, respectively, using the relevant peak areas of the labelled and unlabelled molecular peaks observed in their high-resolution mass spectra^43^ (see ESI, Fig. S8†).

### Probing the pH dependency of G*x*-DiMan–DC-SIGN/R binding *via* GNP fluorescence quenching

GNPs are universal, outstanding quenchers for a wide range of fluorophores,^33^ due to their high molar extinction coefficients and broad absorption spectrum.^44^ Moreover, GNP-mediated fluorescence quenching has been found to follow a nanosurface energy transfer (NSET) mechanism, where the QE shows an inverse 4^th^ power dependence on the dye-GNP distance, *d*, *i.e.* QE = 1/[1 + (*d*/*d*_0_)^4^], where *d*_0_ is the distance that gives 50% QE.^45^ As a result, GNP based fluorescence quenching can occur over a much longer distance than organic quenchers relying on the Förster resonance energy transfer (FRET) mechanism, where QE exhibits an inverse 6^th^ power dependence on distance, *R*; *i.e.* QE = 1/[1 + (*R*/*R*_0_)^6^].^46,47^ Therefore, GNP quenchers can be orders of magnitude more efficient than organic quenchers (with quenching efficiency, QE, as high as 99.97% being reported for a closed DNA hairpin system),^48^ making them well-suited for probing MLGIs between dye labelled lectins and glycan-GNPs.^20^

To determine the pH-dependency of MLGI between DC-SIGN/R and G*x*-DiMan, fluorescence spectra were recorded at 0.4 pH unit intervals over a range from 4.6 to 7.4 for Atto-643-labelled DC-SIGN/R in the absence and presence of G*x*-DiMan. A fixed concentration of 5.0 nM for G13-DiMan or 0.50 nM for G27, respectively, was employed to compensate for the much higher extinction coefficient of G27 over G13 (∼10 fold), ensuring that they have similar UV-vis absorbance to minimise any possible difference caused by GNP's inner filter effect (ESI, Fig. S5†).^49^ A fixed protein : G*x*-DiMan molar ratio of 10 : 1 for G13-DiMan or 60 : 1 for G27-DiMan was used for the fluorescence quenching measurement. These ratios were lower than those required to form a closely packed monolayer of DC-SIGN molecules on the G*x*-DiMan surfaces (estimated to be ∼43 and ∼92 for G13-DiMan and G27-DiMan, respectively, based on G*x*-DiMan particle surface areas calculated using their *D*_h_s, and a footprint area of ∼35 nm^2^ per each bound DC-SIGN molecule^7^). Thus, surface saturation should not be a limiting factor for the observed G*x*-DiMan–DC-SIGN binding (and hence fluorescence quenching). All fluorescence measurements were carried out in an MES (2-(*N*-morpholino)ethane sulfonic acid) buffer (25 mM MES, 100 mM NaCl, and 10 mM CaCl_2_), which is well-suited for buffering within the pH range of 4.6 to 7.4.^36,50^ The fluorescence of Atto-643 dye alone was found to be independent of pH over pH 4.6 to 7.4 (see ESI, Fig. S9†), in good agreement with the product information.^42^ Therefore, any fluorescence responses observed as a function of pH for DC-SIGN/R or G*x*-DiMan + DC-SIGN/R samples must come from the conformational changes of DC-SIGN/R and/or their binding interactions with G*x*-DiMan, and not from the dye itself. The fluorescence spectra of DC-SIGN/R alone and DC-SIGN/R + G*x*-DiMan samples at a variety of pHs are provided in the ESI, Fig. S9 and S10.† The integrated fluorescence from 650 to 800 nm was used to calculate the quenching efficiency (QE) in the presence of G*x*-DiMan over that in the absence of G*x*-DiMan *via*eqn (1) below:1
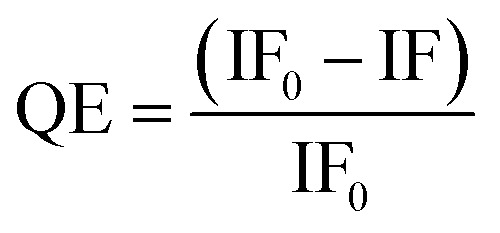
where IF_0_ and IF are integrated fluorescence of DC-SIGN/R in the absence and presence of G*x*-DiMan, respectively.

**Fig. 2:**
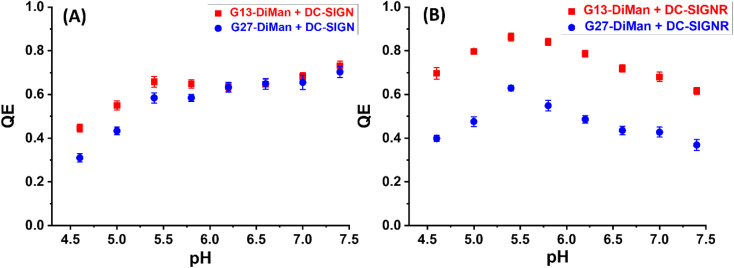
Fluorescence quenching efficiency (QE) – pH relationships for G13-DiMan (5.0 nM) or G27-DiMan (0.50 nM) binding with DC-SIGN (A) or DC-SIGNR (B) over a pH range of 4.6 to 7.4 at a fixed lectin : G*x* molar ratio of 10 : 1 for G13-DiMan or 60 : 1 for G27-DiMan.

Assuming that G*x*-DiMan bound lectins are fully quenched (indeed, QEs as high as 99.97% were reported for GNP quenched fluorophores^48^), then the QE here represents the percentage of added lectins that are bound to G*x*-DiMan. Therefore, QE is positively correlated with the MLGI affinity between G*x*-DiMan and DC-SIGN/R (*i.e.*, the higher the affinity, the higher the QE). Unfortunately, the much stronger inner filter effect of the bigger G13/G27 over G5 (with absorption extinction coefficients of ∼2.3 × 10^8^ and 2.4 × 10^9^ M^−1^ cm^−1^*vs.* 1.1 × 10^7^ M^−1^ cm^−1^ for G5) has prevented us from being able to accurately measure their MLGI affinities (*K*_d_s) using the GNP fluorescence quenching assay established with the G5-glycan probes.^20^ In that case, the apparent *K*_d_s were derived by fitting the QE – concentration relationships of 1 : 1 molar mixed lectin : G5-glycan samples *via* the Hill equation, where accurate QEs over a suitable concentration range (*e.g.*, sub-nM to tens of nM) were required even for strong MLGIs with low nM *K*_d_s.^20^ This has become unfeasible for G13/G27-DiMan because of their very strong absorption. Therefore, QE was employed as a qualitative representation of G*x*-DiMan–DC-SIGN/R MLGI affinity here. The resulting QE *vs.* pH plots for DC-SIGN/R binding with G*x*-DiMan are shown in Fig. 2.

Both the QEs for DC-SIGN/R binding with G*x*-DiMan were found to be strongly dependent on pH, but with notable differences (Fig. 2). For DC-SIGN, its binding affinity (represented by QE) was high and remained almost constant as pH was reduced from 7.4 (normal physiological pH) to 5.4, and further reduction of pH led to significantly reduced QE (Fig. 2A). In contrast, the QE for DC-SIGNR displayed an inverse V-shaped response with pH; it peaked at ∼pH = 5.4 and then decreased with the increasing deviation from this point (with pH going either higher or lower). Changing the GNP scaffold size (G13 *vs.* G27) did not significantly affect the trend of their QE – pH relationships. DC-SIGNR appeared to bind progressively more strongly (displaying higher QE) with G*x*-DiMan as pH was reduced from 7.4 to 5.4, suggesting no pH triggered release of bound glycan ligands for DC-SIGNR over this pH range. This result is consistent with an earlier report that DC-SIGNR does not release its ligands at earlier endosomal pHs (∼6.0).^28^ DC-SIGNR binding was found to be weakened as pH was reduced further to <5.4, suggesting that it may release ligands in late endosomes (pH ∼ 5.0)^27^ or lysosomes (pH ∼ 4.6). DC-SIGN, on the other hand, may bind ligands stably over the normal physiological to early endosomal pH range (*i.e.*, pH 7.4 to ∼5.8), which then readily releases them as pH is further reduced.

### Probing pH-dependency of DC-SIGN/R–G*x*-DiMan assemblies by dynamic light scattering

**Fig. 3:**
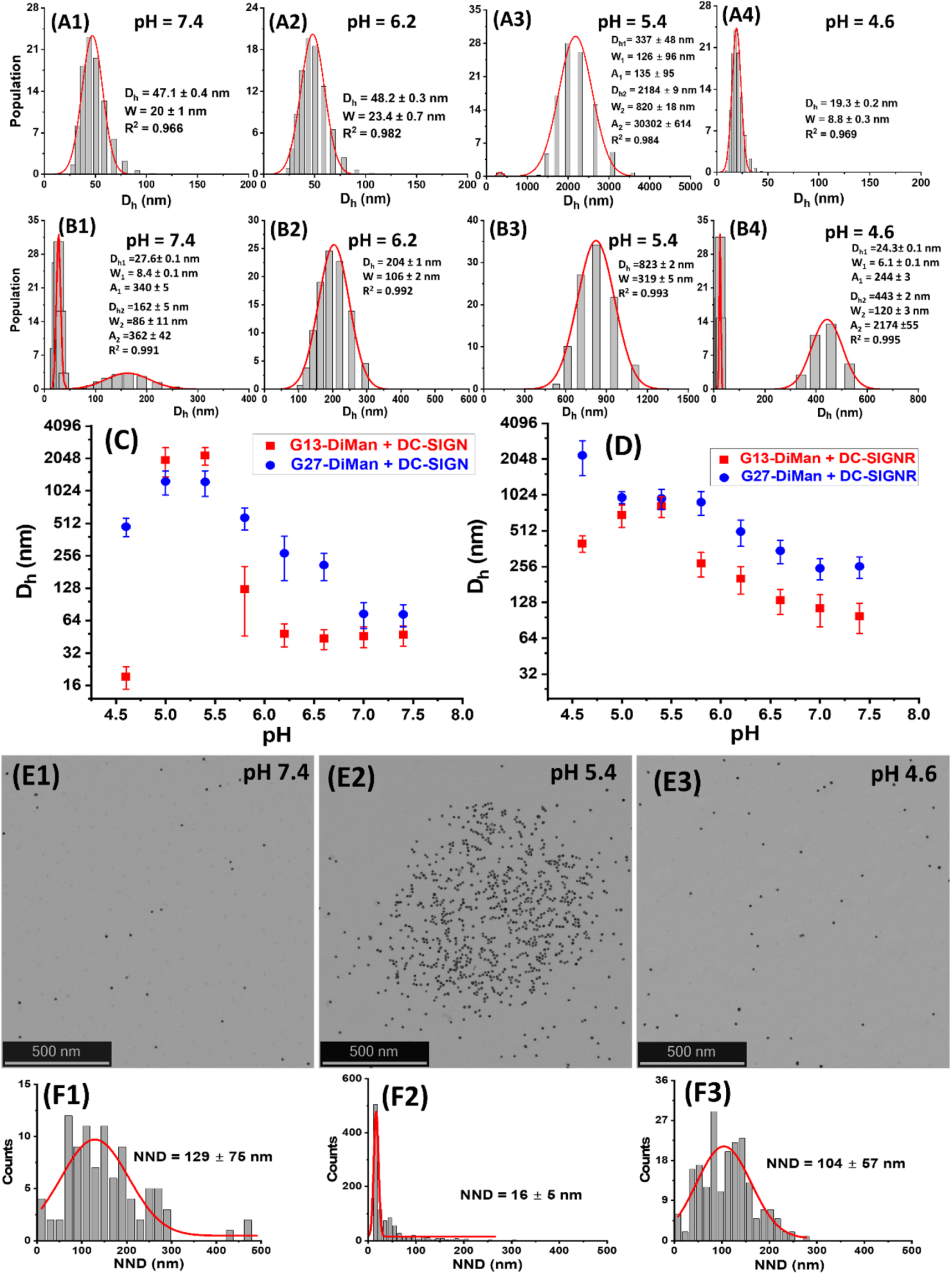
(A and B) Representative *D*_h_ distribution histograms for DC-SIGN (50 nM, A1–A4) or DC-SIGNR (50 nM, B1–B4) binding with G13-DiMan (5.0 nM) at a systematically decreasing pH from 7.4 to 4.6. (C and D) Comparison of the *D*_h_ – pH relationships for DC-SIGN (C) and DC-SIGNR (D) binding with G13-DiMan (red) or G27-DiMan (blue). The *D*_h_ is displayed as mean *D*_h_ ± ½FWHM (full-width at half-maximum of the Gaussian fit). For those showing two distribution histograms, the mean *D*_h_ and mean FWHM were obtained from the linear addition of their relative abundances: *i.e.*, mean *D*_h_ = (*xc*_1_*A*_1_%) + (*xc*_2_*A*_2_%); mean FWHM = (*w*_1_*A*_1_%) + (*w*_2_*A*_2_%). (E and F) Representative STEM images (E1–E3) and the corresponding nearest neighbour distance (NND) histograms fitted using single Gaussian distribution functions (F1–F3) of cryo-prepared G13-DiMan (5 nM) + DC-SIGN (50 nM) samples at different pHs: 7.4 (E1 and F1); 5.4 (E2 and F2), and 4.6 (E3 and F3).

To probe how pH may affect the MLGI properties between G*x*-DiMan and DC-SIGN/R, we have further monitored the hydrodynamic diameters (*D*_h_s) of the resulting lectin–GNP complexes by dynamic light scattering (DLS). Similarly, all measurements were performed at a fixed lectin (wild-type, no dye labelling) : G*x*-DiMan molar ratio of 10 : 1 and 60 : 1 for G13-DiMan (5.0 nM) and G27-DiMan (0.50 nM), respectively, in the MES buffer at 0.4 pH unit intervals from pH 4.6 to 7.4. The *D*_h_s of the G*x*-DiMan conjugates or DC-SIGN/R alone were found to be stable and showed no significant changes over this pH range (ESI, Fig. S11 and S12†); therefore any notable changes in *D*_h_ observed for the G*x*-DiMan + lectin samples must be caused by their specific binding or unbinding. Representative *D*_h_ distribution histograms (volume population) for DC-SIGN/R binding with G13-DiMan at pH 7.4, 6.2, 5.4 and 4.6 are shown in Fig. 3A1–4 and B1–4, respectively (see ESI, Fig. S13–S16† for all other pHs). The corresponding average *D*_h_ – pH relationships of DC-SIGN/R binding with G*x*-DiMan (*x* = 13 and 27) are shown in Fig. 3C and D, respectively.

As shown in Fig. 3A and B, the *D*_h_ dependencies on pH for binding induced lectin–G*x*-DiMan complexes were very different between DC-SIGN and DC-SIGNR. For DC-SIGN, it formed compact complexes at a normal physiological pH of 7.4 with *D*_h_s (mean ± ½FWHM) of ∼47 ± 10 nm and ∼73 ± 17 nm with G13-DiMan and G27-DiMan, respectively. These *D*_h_s match well to those expected for single G*x*-DiMan particles bound with a single layer of DC-SIGN molecules. This result is fully consistent with our previous observation that DC-SIGN binds tetravalently with all four CRDs to a single G5-glycan to form compact isolated G5-lectin complexes.^7,20^ This binding mode for DC-SIGN was maintained from pH 7.4 to 6.2 in binding to G13-DiMan (ESI, Fig. S13†) or from pH 7.4 to 7.2 in binding to G27-DiMan (ESI, Fig. S15†), although reducing pH to below such ranges produced significantly larger *D*_h_ sizes, likely due to a change of binding mode for DC-SIGN. For example, reducing pH to 5.8 produced two distinct *D*_h_ species for the G13-DiMan + DC-SIGN sample, a small and narrowly distributed species (*D*_h_ ∼ 35 ± 8 nm) and a large and broadly distributed species (*D*_h_ ∼ 200 ± 135 nm). The former *D*_h_ is slightly smaller than that at pH 7.4 (∼47 ± 10 nm) but larger than that of G13-DiMan alone (∼19 ± 5 nm), suggesting that it is likely to consist of a single G13-DiMan bound with fewer DC-SIGN molecules than that at pH 7.4. In contrast, the latter *D*_h_ is too big to be single-layer lectin coated individual G13-DiMan particles and is most likely to be large-scale G13-DiMan–DC-SIGN assemblies formed by crosslinking (ESI, Fig. S11E†). This result indicated that a change in DC-SIGN binding mode from simultaneous tetravalent binding to single G13-DiMan to crosslinking occurred at this pH, presumably *via* pH-induced conformational changes in DC-SIGN (since the *D*_h_ of G*x*-DiMan or DC-SIGN alone did not change over pH 4.6 to 7.4). This conclusion was further supported by the observation that the *D*_h_ distribution histograms of the DC-SIGN + G13-DiMan sample at pH 5.8 closely resembled that of the DC-SIGNR + G13-DiMan sample at pH 7.4 (Fig. 3B1), where DC-SIGNR was known to crosslink with GNP-glycans.^20^ Reducing pH to 5.4 and 5.0 produced only large DC-SIGN–G13-DiMan complexes with *D*_h_s of ∼400 nm and ∼2 μm for the minor and major species, respectively (note here that the absolute *D*_h_ values of the large lectin–GNP assemblies were not accurate as they formed unstable dispersions and grew with time and eventually precipitated out of solution, see ESI, Fig. S13†). This result suggests that DC-SIGN has completely adopted the crosslinking binding mode in this pH range, leading to the formation of large-scale, extensively crosslinked DC-SIGN–G13-DiMan complexes. Interestingly, reducing pH further to 4.6 yielded only one small and narrowly distributed species with a *D*_h_ (*e.g.*, ∼19 ± 5 nm) identical to that of G13-DiMan alone (Fig. 3A4), indicating that all bound DC-SIGN molecules were dissociated from G13-DiMan at pH 4.6.

The adoption of different binding modes for DC-SIGN at different pHs was further verified by performing “cryo-snapshot” scanning/transmission electron microscopy (S/TEM) imaging^7,20,51^ of the G13-DiMan + DC-SIGN samples at three different pHs, 7.4, 5.4 and 4.6. This was performed by rapid plunge freezing the sample in liquid ethene, followed by vacuum drying before being loaded onto a TEM grid for S/TEM imaging. We have shown previously that this technique can capture the native dispersion states of nanoparticles in solution.^52^ The resulting STEM images revealed that the G13-DiMan + DC-SIGN samples prepared at pH 7.4 and 4.6 existed mainly as isolated single GNP particles with large and broadly distributed nearest neighbour distances (*e.g.*, NND = 129 ± 75 nm and 107 ± 57 nm, see Fig. 3E1/E3 and F1/F3), while those prepared at pH 5.4 existed mainly as large scale, clustered GNP assemblies of ∼1 μm across with a small and narrowly distributed NND (∼16 ± 6 nm, see Fig. 3E2/F2). These results were fully consistent with their *D*_h_ sizes measured by DLS (Fig. 3), confirming that the G13-DiMan + DC-SIGN samples at pH 7.4 and 4.6 indeed existed as isolated single GNP particles (with or without coating of a lectin monolayer) while those at pH 5.4 were mainly made of extensively crosslinked GNP–lectin complexes. Consistent with their distinct *D*_h_ sizes, the G13-DiMan + DC-SIGN samples exhibited very different colloidal stabilities at different pHs as expected. The two samples with large *D*_h_s (pH 5.0 and 5.4) were found to have precipitated out of the solution, while those with small *D*_h_s (*i.e.*, pH 4.6 and 6.2–7.4) remained well dispersed and showed no signs of colour change or precipitation after overnight incubation (see ESI, Fig. S13I†). Moreover, based on the much weaker affinity of DC-SIGNR over DC-SIGN in binding to G5-DiMan observed at pH 7.4,^7,20^ despite their identical monovalent CRD–mannose binding motifs,^30^ changing DC-SIGN binding mode from simultaneous tetravalent binding to single GNP-DiMan to crosslinking would thus expect to weaken its G13-DiMan affinity at ≤pH 5.8. This is exactly what has been observed in the fluorescence quenching experiments described in the previous section (Fig. 2A).

For DC-SIGNR, the average *D*_h_s of its complexes with G*x*-DiMan were found to generally increase with the decreasing pH from 7.4 to 4.6, although significant differences in the distribution species were also noticeable. For example, small species corresponding to individual G13-DiMan particles and large species corresponding to crosslinked DC-SIGNR–G13-DiMan assemblies were found to coexist at pH 7.4. This was likely due to the relatively weak binding affinity of DC-SIGNR at this pH that made it unable to fully crosslink all G13-DiMan particles (Fig. 3B1). As pH was reduced, the amounts of crosslinked species increased while that of isolated single particle species decreased. As pH was lowered to 6.2 and below, only a single large crosslinked species was observed (from pH 6.2 to 5.0), indicating highly efficient DC-SIGNR–G13-DiMan crosslinking. This result was consistent with the strong affinity of DC-SIGNR for binding to G13-DiMan (high QE) over this pH range observed in the QE experiment (Fig. 2B). Further reducing pH to 4.6 produced two different *D*_h_ species, signifying partial dissociation of DC-SIGNR–G13-DiMan complexes (Fig. 3B, and ESI, Fig. S14†). This result was also consistent with their reduced affinity (reduced QE, see Fig. 2B) at this pH. Overall, the *D*_h_ – pH dependence observed for G13-DiMan–DC-SIGNR binding was consistent with that expected for their inverted V-shaped QE – pH dependence observed in the fluorescence quenching experiment, where DC-SIGNR was found to display the highest affinity (QE) at pH 5.4. In general, the average *D*_h_s of DC-SIGNR–G*x*-DiMan complexes were found to be significantly greater than that expected for single G*x*-DiMan particles coated with a single layer of DC-SIGNR molecules, especially for G27-DiMan, indicating that they were formed through G*x*-DiMan–DC-SIGNR crosslinking. These results matched well to what was expected for DC-SIGNR, based on its crosslinking binding mode with G5-DiMan observed previously at pH 7.4.^20^

Interestingly, the consistently small *D*_h_s for G13-DiMan–DC-SIGN complexes observed over pH 7.4 to 6.2 also matched well to their consistently stable QE over this pH range (Fig. 2A). Moreover, the large *D*_h_ in the more acidic pH environment (*e.g.*, 5.4 to 5.0) also correlated with the reduced fluorescence QEs observed for DC-SIGN at such pHs, while the dissociation of DC-SIGN at pH 4.6 gave the lowest QE. The *D*_h_ and fluorescence quenching data obtained here suggest that low pH environments are likely to cause conformational changes in DC-SIGN/R and/or alter their binding modes, reducing their MLGI affinities to trigger the release of bound glycan ligands. Our results thus suggested that acidic intracellular endosomes or lysosomes may cause glycan ligands to be released from their DC-SIGN/R complexes during the natural trafficking processes. The different pH-dependent MLGI behaviours in solution between DC-SIGN and DC-SIGNR observed here may be associated with their different functions in the transmission of virus infections,^53^ although further studies under conditions that mimic more closely those of DC-SIGN/R-virus interactions on cell surfaces are still needed.

### pH-dependent switching of DC-SIGN/R–G*x*-DiMan complexes

**Fig. 4:**
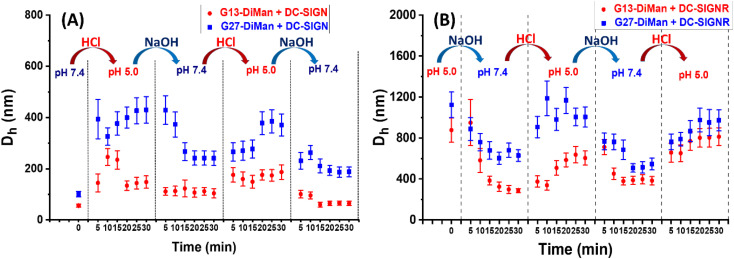
Summary of the *D*_h_ – time dependence plots for DC-SIGN (A) or DC-SIGNR (B) binding with G*x*-DiMan upon cycling the buffer pH between 7.4 and 5.0. *D*_h_s are displayed as mean *D*_h_ ± ½FWHM obtained from the Gaussian fits. For samples displaying two distinct peaks, mean *D*_h_ = (*xc*_1_*A*_1_%) + (*xc*_2_*A*_2_%); and mean FWHM = (*w*_1_*A*_1_%) + (*w*_2_*A*_2_%).

To check whether DC-SIGN/R binding with G*x*-DiMan is reversible, the *D*_h_s of G*x*-DiMan + DC-SIGN/R complexes were monitored by switching the solution pH between pH 7.4 and 5.0 by cyclic addition of NaOH or HCl. First, calibration curves were constructed to determine the amount of HCl (1.0 M) or NaOH (1.0 M) required to change the MES buffer pH to the required value. Then, the *D*_h_s of the G*x*-DiMan + DC-SIGN/R samples were measured by cycling the buffer pH between 7.4 and 5.0, *via* the alternate addition of NaOH and then HCl in four pH cycles. The kinetics of the pH switching was followed by measuring *D*_h_ every 5 min until it was stabilised. The resulting *D*_h_ distribution histograms are given in ESI, Fig. S17–S32†, and the *D*_h_ – time responses upon pH cycling between 7.4 and 5.0 are summarised and shown in Fig. 4.

Fig. 4 reveals that, upon switching pH from 7.4 to 5.0, there was a rapid increase in the *D*_h_s for both DC-SIGN/R–G*x*-DiMan complexes, while switching pH back from 5.0 to 7.4 led to a rapid decrease in the *D*_h_. This result was fully consistent with that observed in the previous section (Fig. 3), which also gave much larger lectin–G*x*-DiMan assemblies at pH 5.0 than at pH 7.4, especially for DC-SIGN (Fig. 3). The small *D*_h_ at pH 7.4 was fully consistent with DC-SIGN's tetravalent binding with single G5-DiMan at pH 7.4 as reported previously.^6,20^ The pH-dependent *D*_h_ switching between pH 5.0 and 7.4 was partially reversible for both DC-SIGN and DC-SIGNR, and that with G13-DiMan appeared to have better reversibility than that with G27-DiMan, especially with DC-SIGN. In all cases, the *D*_h_ values of the lectin–G*x*-DiMan assemblies were stabilised at ∼30 min after each pH switching, in both the pH down and up directions. This result indicated that 30 min was required to achieve the desired lectin–G*x*-DiMan assemblies or dis-assemblies under our experimental conditions in solution. This result is also consistent with DC-SIGN's biological role as an endocytic and recycling receptor:^28^ it binds strongly to endocytose target ligands at normal physiological pH and then releases the ligands under the acidic environment of early to late endosomes so that it can be recycled back to the cell surface for further binding and endocytosis of more ligands. It should be noted that while switching pH from 5.0 to 7.4 generally produced smaller assemblies (smaller *D*_h_s), the stabilised *D*_h_s for the DC-SIGN/R–G*x*-DiMan complexes were still larger than *D*_h_s for those directly prepared at pH 7.4 (especially for G27-DiMan). This indicated that only partial restoration of the original G*x*-DiMan–DC-SIGN complexes was achieved within 30 min.

## Conclusion

In conclusion, we have probed the pH-dependent MLGI properties between DC-SIGN/R and G*x*-DiMan *via* fluorescence quenching and hydrodynamic size studies for the first time. We have revealed that both DC-SIGN/R binding with G*x*-DiMan are strongly pH-dependent and partially reversible. Our pH-dependent fluorescence quenching studies show that DC-SIGN binds strongly and stably with G*x*-DiMan from neutral to weakly acidic pH (*e.g.*, 7.4 to 5.4, comparable to the normal physiological to endosomal pH range), but its binding is significantly reduced at lower pH. This result correlates well with DC-SIGN's biological function as an endocytic and recycling receptor,^28^ which requires it to bind and endocytose target ligands at normal physiological pH and subsequently release them under the acidic environment of intracellular endosomes for receptor recycling. In contrast, DC-SIGNR exhibits the strongest binding (highest QE) with G*x*-DiMan at pH 5.4, and any deviation from this pH leads to progressively reduced affinity (lower QE). This result suggests that DC-SIGNR bound ligands may not be released in early endosomes. Instead, they are likely to be released in the more acidic environment of late endosomes or lysosomes. Our DLS data have suggested that DC-SIGN/R adopt their characteristic binding modes at pH 7.4 (*i.e.*, tetravalent binding with all four CRDs to a single G*x*-DiMan for DC-SIGN and crosslinking with different G*x*-DiMans for DC-SIGNR),^7,20^ but their binding modes are mainly shifted to crosslinking as pH is reduced, leading to the formation of larger DC-SIGN/R–G*x*-DiMan complexes, and reducing pH further to 4.6 leads to the complete dissociation of the G13-DiMan–DC-SIGN complexes. We have further revealed that both DC-SIGN/R bindings with G*x*-DiMan are partially reversible in a pH-dependent manner. Overall, DC-SIGN binding with glycan ligands is weakened at weakly acidic pH, but the affinity is reinstalled as pH is switched back to 7.4, consistent with DC-SIGN's role as a recycling endocytic receptor.^28^ In addition, DC-SIGNR's strong affinity for G*x*-DiMan over pH 5.4–5.8 indicates that it does not release ligands at earlier endosomal pH, making it difficult to act as a ligand recycling receptor.^28^ This work thus reveals a new insight into DC-SIGN/R's distinct pH-dependent MLGI behaviours in solution at the molecular level, which are fundamental for their biological functions. A potential limitation of this study is that all DC-SIGN/R pH-dependent MLGI studies are performed in solution, not in their native cell membrane environment, using the G*x*-DiMan probes that also have different size and glycan patterns from true viruses. The use of cell membrane immobilised DC-SIGN/R and pseudo-viruses to mimic more closely the natural DC-SIGN/R-virus interactions is still needed, which is on our agenda and will be reported in the following paper.

## Experimental section

### Materials

Gold(iii) chloride trihydrate, sodium hydroxide, trisodium citrate, copper sulphate, sodium sulphate, calcium chloride, HEPES, lipoic acid (LA), sodium ascorbate, tris[(1-benzyl-1*H*-1,2,3-triazol-4-yl)methyl]amine (TBTA), methanol, ethanol, chloroform, phenol, bovine serum albumin, tetrahydrofuran, tris-(hydroxymethyl)aminomethane (tris base), hydrochloric acid, sodium chloride, MES (2-(*N*-morpholino)ethane sulfonic acid), ethylenediamine tetraacetic acid, and guanidine hydrochloride were purchased commercially from Sigma-Aldrich, Alfa Aesar, Fluorochem, and Thermo Scientific with >99% impurity and used as-received without further purification unless specified elsewhere. Thiol-reactive Atto-643 dye was commercially obtained from ATTO-Tech GmbH. Ultrapure water (resistance > 18.2 MΩ cm), purified using an ELGA Purelab classic UVF system, was used for all experiments and making buffers.

### Synthesis of 13 nm gold nanoparticles^36^

Freshly prepared aqueous solution of gold(iii) chloride trihydrate (1.0 mM, 400 mL) was placed in a 500 mL three-necked round-bottomed flask and the solution was then heated to reflux in a 130 °C oil bath under stirring. When the solution began to reflux, trisodium citrate solution (38 mM, 40 mL) was quickly added. The solution colour quickly turned from yellow to wine red in ∼1 min, indicating the formation of GNPs. The reaction was further refluxed under magnetic stirring for another 1 h to ensure that the reaction was complete. The GNP solution was then removed from the oil bath and was allowed to cool down to RT naturally under stirring. Then, the GNP solution was transferred to a clean glass container and stored at RT until use. This produced citrate stablised 13 nm GNPs (G13) with a mean diameter of ∼13 nm according to the TEM images.

### Synthesis of 27 nm gold nanoparticles^36^

Freshly prepared aqueous solution of gold(iii) chloride trihydrate (0.25 mM, 400 mL) was placed in a 500 mL two-necked round-bottomed flask, and NaOH (1.0 mM, 50 mL) was then added directly into the solution. The mixture was stirred for 30 min and then heated to reflux in a 130 °C oil bath under magnetic stirring. After the solution started to reflux, trisodium citrate solution (166 mM, 6 mL) was then quickly added. The solution colour gradually changed from yellow to light red in 15 min. The reaction was refluxed for another 1 h to complete the synthesis. The solution was then taken out of the oil bath and kept stirring for 1 h until it was cooled down to RT. This produced 27 nm GNPs (G27) stock, which was transferred to a clean glass container and stored at room temperature until use.

### Synthesis of LA–EG_4_–DiMan^20,40^

LA–EG_4_–C≡CH (50 mg, 0.120 mmol), 1-azido-3,6-dioxaoct-8-yl-α-d-mannopyranosyl-(1→2)-α-d-mannopyranoside, N_3_–EG_2_–DiMan (66 mg, 0.132 mmol), CuSO_4_·5H_2_O (1.1 mg, 0.0043 mmol), TBTA (4.0 mg, 0.0075 mmol), and sodium ascorbate (3.2 mg, 0.0162 mmol) were dissolved in 2 mL of THF/H_2_O (1 : 1, vol/vol). The resulting solution was stirred overnight at room temperature in darkness. The next day, the consumption of all starting compounds was confirmed by thin layer chromatography (TLC). The solvent was then evaporated, and the desired ligand was purified by size exclusion chromatography using a Biogel P2 column using ammonium formate as an eluent. The fractions containing the desired pure ligand were combined and lyophilised to give the desired ligand as a yellow solid (93.4 mg, 0.101 mmol, 77% yield). TLC: (CHCl_3_/MeOH 3 : 1) *R*_f_ 0.57; ^1^H NMR (400 MHz, D_2_O) *δ* (ppm): 8.10 (s, 1H), 5.12 (s, 1H), 5.03 (s, 1H), 4.73–4.60 (m, 3H), 4.08 (s, 1H), 3.99 (dd, 3H, *J* = 10.2, 5.1 Hz), 3.94–3.82 (m, 5H), 3.69 (dt, 31H, *J* = 12.8, 7.1, 6.7 Hz), 3.45–3.30 (m, 2H), 3.30–2.33 (m, 2H), 2.26 (t, 2H, *J* = 7.3 Hz), 1.99 (dt, 1H, *J* = 12.9, 6.9 Hz), 1.78–1.54 (m, 4H), 1.42 (q, 1H, *J* = 7.6 Hz); ^13^C NMR (100 MHz, D_2_O) *δ* (ppm): 176.7, 144.1, 125.5, 102.2, 98.3, 78.6, 73.2, 72.7, 70.2, 70.1, 69.9, 69.6, 69.6, 69.5, 69.5, 69.4, 69.2, 68.9, 68.8, 68.7, 66.9, 66.8, 66.5, 66.5, 63.2, 63.1, 61.4, 61.1, 60.8, 59.3, 56.5, 50.1, 50.0, 46.6, 40.2, 38.9, 38.1; LC-MS: calculated *m*/*z* for C_37_H_66_N_4_O_18_S_2_ (M + H)^+^ 919.38, found 919.78.

### Preparation of G*x*-DiMan^20^

Twenty mL each of the citrate stabilised G13 or G27 stock solutions were directly added to the required amount of LA–EG_4_–DiMan ligand stock solution (in water) at a GNP : ligand molar ratio of 1 : 3000 for G13 or 1 : 10 000 for G27. The resulting solutions were magnetically stirred at room temperature in the dark overnight to promote the ligand exchange *via* gold–thiol self-assembly. After that, the resulting mixtures were divided into 1.5 mL portions into Eppendorf tubes and centrifuged at 17 000 × *g* for 30 min for G13-conjugates or 6000 × *g* for 15 min for G27-conjugates to remove any unbound free ligands. After careful withdrawal of the clear supernatant, the GNP residues were washed with pure water (3 × 500 μL for each tube) followed by centrifugation three times to remove any unbound free ligands. For G27, the Eppendorf tubes were pre-washed with 0.025% Tween-20 aqueous solution before being used in G*x*-DiMan purification to prevent GNP sticking to the Eppendorf walls. The G*x*-DiMan concentrations were determined using the Beer–Lambert law using their peak absorbance at ∼520 nm and molar extinction coefficient of 2.32 × 10^8^ and 2.39 × 10^9^ M^−1^ cm^−1^ for G13 and G27, respectively (see ESI, Fig. S4 and S5†).

### Protein production and labelling^7,20^

The soluble extracellular segments of DC-SIGN and DC-SIGNR were expressed as inclusion bodies in *E. coli* and purified using a mannose–Sepharose affinity column followed by a Superdex size exclusion column as reported previously.^6,7^ The mutant proteins, DC-SIGN Q-274C and DC-SIGNR R278C, were constructed by site-directed mutagenesis and labelled with Atto-643 maleimide as described previously.^20^ The labelled proteins were purified using mannose–Sepharose affinity columns. All the proteins were characterized by high-resolution mass spectroscopy (HRMS, see ESI, Fig. S6 and S8†). The dye labelling efficiency (per protein monomer) was determined to be ∼82% and ∼90% for DC-SIGN and DC-SIGNR, respectively, based on the relative peak areas of the labelled and unlabelled protein peaks measured by HR-MS^43^ (ESI, Fig. S8†).

### Fluorescence spectra^20^

All fluorescence spectra were recorded on a Horiba FluoroMax-4 Spectro-fluorometer using a 0.70 mL quartz cuvette with an optical path length of 1 cm at a fixed excitation wavelength (*λ*_ex_) of 630 nm. Emission spectra over 650–800 nm were collected with excitation and emission slit widths of 5 nm at a slow scan speed. All measurements were carried out in a MES buffer (25 mM MES, 100 mM NaCl, and 10 mM CaCl_2_, pH varied from 7.4 to 4.6) containing 1 mg mL^−1^ BSA to minimise any non-specific interactions and absorption to cuvette walls. The required amounts of G*x*-DiMan and DC-SIGN/R were mixed in the MES buffer and incubated at RT for 20 min before their fluorescence spectra were recorded. The fluorescence spectra from 650 to 800 nm were integrated and used to calculate the fluorescence quenching efficiency (QE).

### Dynamic light scattering (DLS).^20^

All measurements were performed on a Malvern Zetasizer NanoZS DLS system at room temperature using disposable polystyrene cuvettes at a sample volume of 400 μL. The hydrodynamic diameters (*D*_h_s, volume populations that were directly provided using the DLS software) of wild-type DC-SIGN/R and G*x*-DiMan were measured in a binding buffer (20 mM HEPES, 100 mM NaCl, 10 mM CaCl_2_, pH 7.8), while their pH-dependent binding studies were performed in an MES buffer (25 mM MES, 100 mM NaCl, 10 mM CaCl_2_, pH varied from 7.4 to 4.6 at 0.4 pH intervals). Each DLS measurement was performed in ten consecutive runs, each lasting 120 seconds, and the average of the ten runs was used to determine the *D*_h_ volume distribution. Each sample was analysed in triplicate, and the resulting averaged *D*_h_ distribution histograms (volume populations in linear scales) were fitted using the standard Gaussian function (single or multiple, depending on the data) using the Origin software (version 2023b) to determine the mean *D*_h_, full-width at half-maximum (FWHM) and polydispersity index (PDI = (FWHM/mean *D*_h_)^2^).^54^ For samples displaying two distinct distribution peaks, a linear addition of their relative abundances was used to calculate their mean *D*_h_ and mean FWHM: *i.e.*, mean *D*_h_ = (*xc*_1_*A*_1_%) + (*xc*_2_*A*_2_%); mean FWHM = (*w*_1_*A*_1_%) + (*w*_2_*A*_2_%).^20^

### Scanning transmission electron microscopy (STEM) imaging^52^

G13-DiMan (5 nM) and DC-SIGN (50 nM) were mixed and incubated for 30 min at room temperature in a MES buffer (25 mM MES, 100 mM NaCl, 10 mM CaCl_2_) at three different pHs, 7.4, 5.4 and 4.6. Then samples were prepared by rapid plunge freezing of a blotted drop (3.5 μL) of each sample, followed by vacuum drying to capture the native dispersion state of the nanoparticles, before being loaded on a TEM grid. STEM images were taken on a Tescan Tensor dedicated 4D-STEM operated at 100 kV using a Dectris direct electron detector as described previously.^20,52^ The STEM images were analysed by using ImageJ 1.4.3.67 software to obtain the nearest neighbour distance (NND) histograms. The mean NNDs were obtained by fitting the distribution histograms with a single Gaussian distribution function as described previously.^20,51^

## Author contributions

R. B., investigation, data curation, formal analysis, writing – original draft; X. N., investigation, formal analysis; D. B., investigation, formal analysis; N. H., investigation, funding acquisition; Y. G., funding acquisition, supervision, methodology, project administration; D. Z., conceptulization, funding acquisition, supervision, methodology, project administration; writing – review & editing.

## Conflicts of interest

There are no conflicts of interest to declare.

## Supplementary Material

NA-006-D3NA01013A-s001
