# Supplementary material for: Probing the pH-dependency of DC-SIGN/R multivalent lectin–glycan interactions using polyvalent glycan-gold nanoparticles
Source: Nanoscale Adv. 2024 Mar 11;6(8):2198–208. doi: 10.1039/d3na01013a (PMC11019501; doi:10.1039/d3na01013a)
Supplement: NA-006-D3NA01013A-s001 [file NA-006-D3NA01013A-s001.pdf]

Electronic Supplementary Material (ESI) for Nanoscale Advances.
This journal is © The Royal Society of Chemistry 2024

ELECTRONIC SUPPORTING INFORMATION (ESI)

Probing pH-Dependency of Multivalent Lectin-Glycan Interactions Using Polyvalent Multifunctional Glycan-Gold Nanoparticles

Rahman Basaran,[1] Xinyu Ning,[1] Darshita Budhadev,[1] Nicole Hondow,[2] Yuan Guo,[3,*] and Dejian Zhou[4,*]

[1] School of Chemistry and Astbury Centre for Structural Molecular Biology, University of Leeds, Leeds LS2 9JT, United Kingdom. Email: d.zhou@leeds.ac.uk.

[2] School of Chemical and Process Engineering, University of Leeds, Leeds LS2 9JT, United Kingdom.

[3] School of Food Science and Nutrition, and Astbury Centre for Structural Molecular Biology, University of Leeds, Leeds LS2 9JT, United Kingdom. Email: y.guo@leeds.ac.uk.

Contents

1. Instrument and Methods S2
2. GNP Synthesis and Characterisation S3-S4
3. Synthesis of the LA-EG_4-DiMan ligands S4-S6
4. GNP-glycan Production and Characterisation S7-S8
5. Determination of Glycan Valency and average inter-glycan distance S8-S9
6. Protein Production and Characterisation S9-S12
    6.1. Wild-Type DC-SIGN and DC-SIGNR Production and Characterisation S9-S10
    6.2. DC-SIGN Q274C-ATTO643 and DC-SIGNR R287C-ATTO643 Production and Characterisation S11-S12
7. Fluorescence Spectra for DC-SIGN, DC-SIGNR, and Atto-643 dye at different pHs S13
8. Fluorescence Spectra for G13/27-DiMan+DC-SIGN/R at different pHs. S14
9. Hydrodynamic Size Measurements of GNP-DiMan + DC-SIGN/R at different pHs S15-S20
10. pH-Switching Studies S21-S36
11. Supporting References S37

1. Instrument and Methods

All moisture-sensitive reactions were performed in oven-dried glassware under a nitrogen atmosphere. Evaporations were carried out at reduced pressure using a Bruker rotary evaporator and a Virtis Benchtop K freeze dryer. Column chromatography was performed using silica gel 60 A, and the progress of the reactions was monitored by thin layer chromatography, TLC, analysis on aluminium sheets pre-coated with silica (Merck Silica Kieselgel 60 F_{254}), then for identifying the compounds present in the reaction mixture, TLC-plates were stained with iodine, orcinol, or *p*-anisaldehyde stains, commercially available. The polar lipoic acid-sugar derivatives were purified by size exclusion chromatography via Biogel P2 column using 20 mM ammonium formate as an eluent to yield the desired pure product.

All ^{1}H and ^{13}C NMR spectra were recorded on a Bruker AV3HD-400 (400 MHz for ^{1}H, 100 MHz for ^{13}C) spectrometer in deuterium oxide (D_2O). All chemical shifts (δs) are denoted in parts per million (ppm) calibrated using residual undeuterated solvents as internal references (D_2O: δ ^{1}H = 4.80 ppm). The coupling constants (*J*) are in parentheses and expressed in Hertz, Hz, and the peak patterns are indicated with the following abbreviations: s = singlet, d = doublet, t = triplet, q = quartet, m = multiplet, dd = doublet of doublets, dt = doublet of triplets. High-resolution mass spectra (HR-MS) were obtained on a Bruker Daltonics MicroTOF mass spectrometer, and deconvoluted mass values (*m/z*) are reported in Daltons, and protein labelling efficiency was also calculated from the ratio of the integral of the labelled protein HR-MS peak to the sum of that of the labelled and unlabelled protein peaks. The mass spectrometry data were also collected using a Bruker HCT Ultra coupled to Ultimate 3000 HPLC (Thermo Scientific). Methanol was used as a solvent to ionise the products; this provides a high-precision detection of ionized samples by classifying the resulting ions by vacuum with respect to mass-charge ratios (*m/z*).

UV-vis absorption spectra were recorded on either a Cary 60 UV-vis spectrophotometer (Agilent Technologies) over 200-800 nm using 1 mL quartz cuvette with an optical path length of 1 cm or on a Nanodrop 2000 spectrophotometer (Thermo Scientific) over the range of 200-800 nm using one drop of the solution with an optical path length of 1 mm. All measurements were done in pure water unless otherwise stated in experimental procedure.

Fluorescence quenching measurements were performed on a Horiba FluroMax 4 spectrometer, using a 0.70 mL quartz cuvette. Centrifugations were performed using a Thermo Scientific Heraeus Fresco 21 and a Beckman Coulter Avanti JXN-30 centrifuge, depending on the speed and volume, at RT unless otherwise noted. Purification or concentration by centrifugation was carried out using Merck Millipore 10 kDa and 30 kDa molecular weight cut-off (MWCO) Amicon Ultra centrifugal filters for protein and GNPs, respectively.

2. GNP Synthesis and Characterisation

The 13 nm and 27 nm GNPs (abbreviated as G13 and G27 respectively) were synthesised in house using a modified literature protocol.[1] Details of the GNP synthesis and purification procedures are given in Experimental Section.

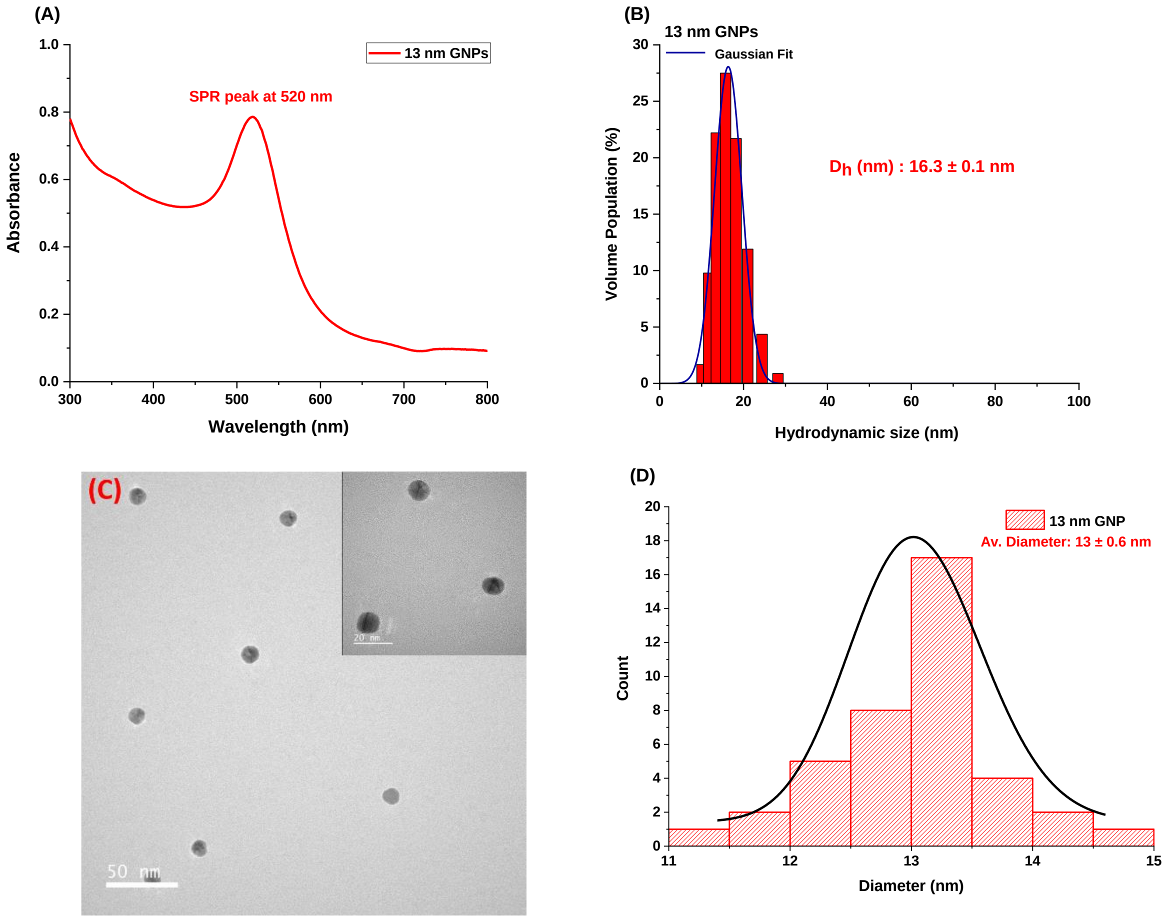

Figure S1. Characterisation of G13. (**A**) UV-vis absorption spectrum of G13 peaks at ~520 nm; (**B**) D_h volume size distribution histogram fitted by Gaussian function, giving a D_h of ~16 nm; (**C**) Typical TEM images of G13 with higher magnification shown as the inset; (**D**) TEM measured G13 diameter histograms fitted by Gaussian, giving a mean size of ~13 nm.

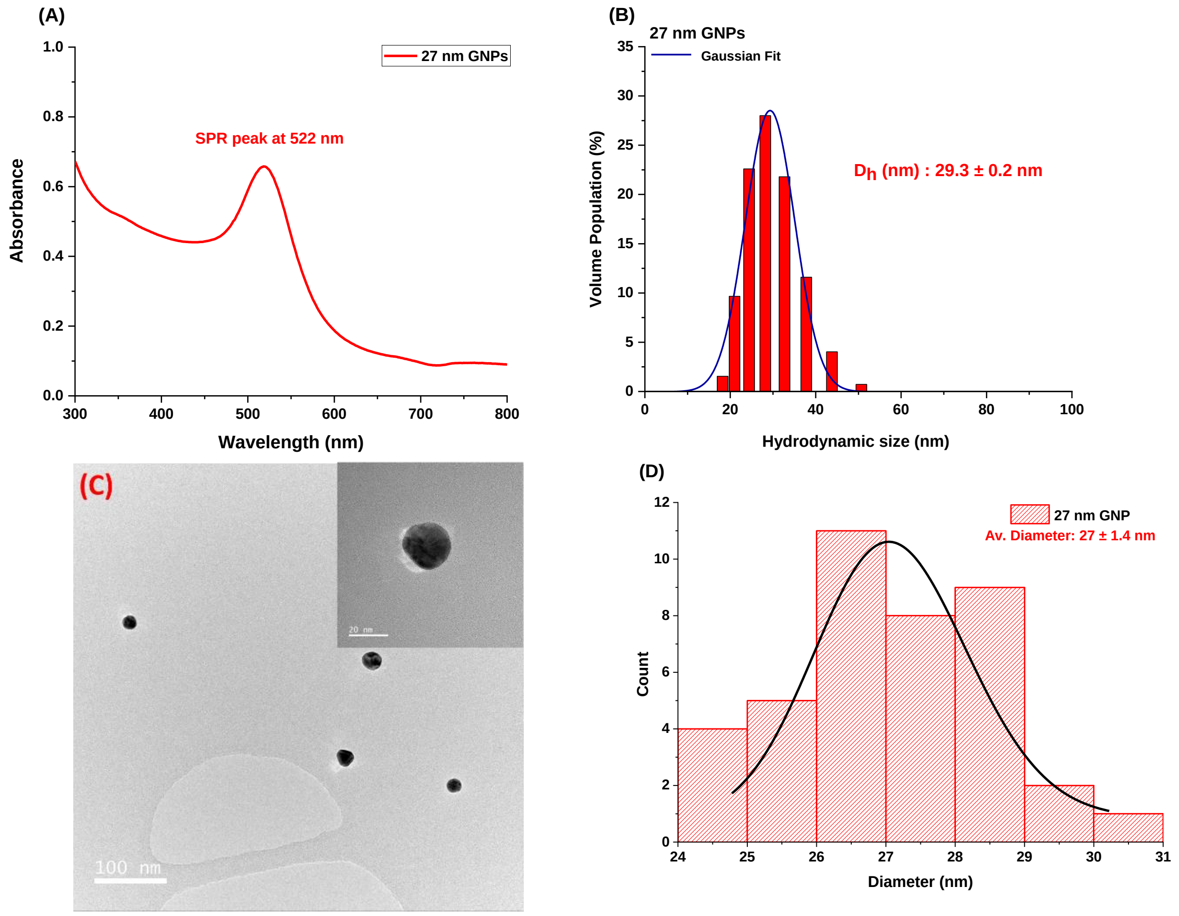

Figure S2. Characterisation of G27. (**A**) UV-vis absorption spectrum of G27 peaks at ~522 nm; (**B**) D_h volume size distribution histogram fitted by Gaussian function, giving a D_h of ~29 nm; (**C**) Typical TEM images of G27 with a higher magnification image shown as the inset; (**D**) TEM measured G27 diameter histograms fitted by Gaussian, giving a mean size of ~27 nm.

3. Synthesis of the LA-EG$_4$-DiMan ligands

LA-EG$_4$-DiMan ligands were synthesised by copper-catalysed "clicking" reaction between LA-EG$_4$-C≡CH and N$_3$-EG$_2$-DiMan as reported previously.[2] The glycans and linkers were synthesised in-house *via* our established protocols. LA-EG$_4$-C≡CH (50 mg, 0.120 mmol), 1-Azido-3,6-dioxaoct-8-yl-α-D-mannopyranosyl-(1→2)-α-D-mannopyraside, N$_3$-EG$_2$-DiMan[3] (66 mg, 0.132 mmol), $CuSO_4.5H_2O$ (1.1 mg, 0.0043 mmol), TBTA (4.0 mg, 0.0075 mmol), and sodium ascorbate (3.2 mg, 0.0162 mmol) were dissolved in 2 mL of THF/H_2O (1:1, vol/vol). The resulting solution was stirred overnight at RT in darkness. Next day, the consumption of all starting compounds was confirmed by TLC. The solvent was then evaporated, and the desired ligand was purified by size exclusion chromatography using Biogel P2 column using ammonium formate as an eluent to obtain the desired product. The reaction scheme of the synthesis is given below. Its ^{1}H, ^{13}C NMR, and LC-MS spectra were shown in Figure S3 below.

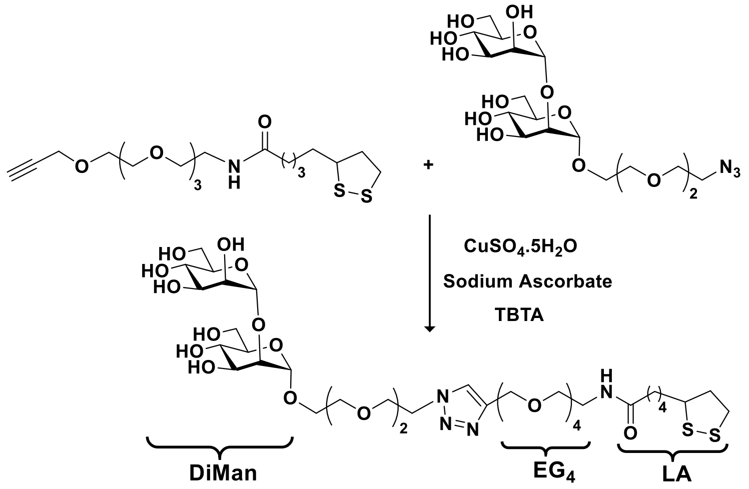

LA-EG_4-DiMan

Yield 77%; TLC: ($CHCl_3$/MeOH 3:1) R_f 0.57; ^{1}H NMR (400 MHz, D_2O) δ (ppm): 8.10 (s, 1H), 5.12 (s, 1H), 5.03 (s, 1H), 4.73 – 4.60 (m, 3H), 4.08 (s, 1H), 3.99 (dd, 3H, *J*=10.2, 5.1 Hz), 3.94 – 3.82 (m, 5H), 3.69 (dt, 31H, *J*=12.8, 7.1, 6.7 Hz), 3.45 – 3.30 (m, 2H), 3.30 – 2.33 (m, 2H), 2.26 (t, 2H, *J*=7.3 Hz), 1.99 (dt, 1H, *J*=12.9, 6.9 Hz), 1.78 – 1.54 (m, 4H), 1.42 (q, 1H, *J*=7.6 Hz); ^{13}C NMR (100 MHz, D_2O) δ (ppm): 176.7, 144.1, 125.5, 102.2, 98.3, 78.6, 73.2, 72.7, 70.2, 70.1, 69.9, 69.6, 69.6, 69.5, 69.5, 69.4, 69.2, 68.9, 68.8, 68.7, 66.9, 66.8, 66.5, 66.5, 63.2, 63.1, 61.4, 61.1, 60.8, 59.3, 56.5, 50.1, 50.0, 46.6, 40.2, 38.9, 38.1; LC-MS: calculated *m/z* for $C_{37}H_{66}N_4O_{18}S_2$ $(M+H)^+$ 919.38, found 919.78.

A- ^{1}H-NMR of LA-EG_4-DiMan

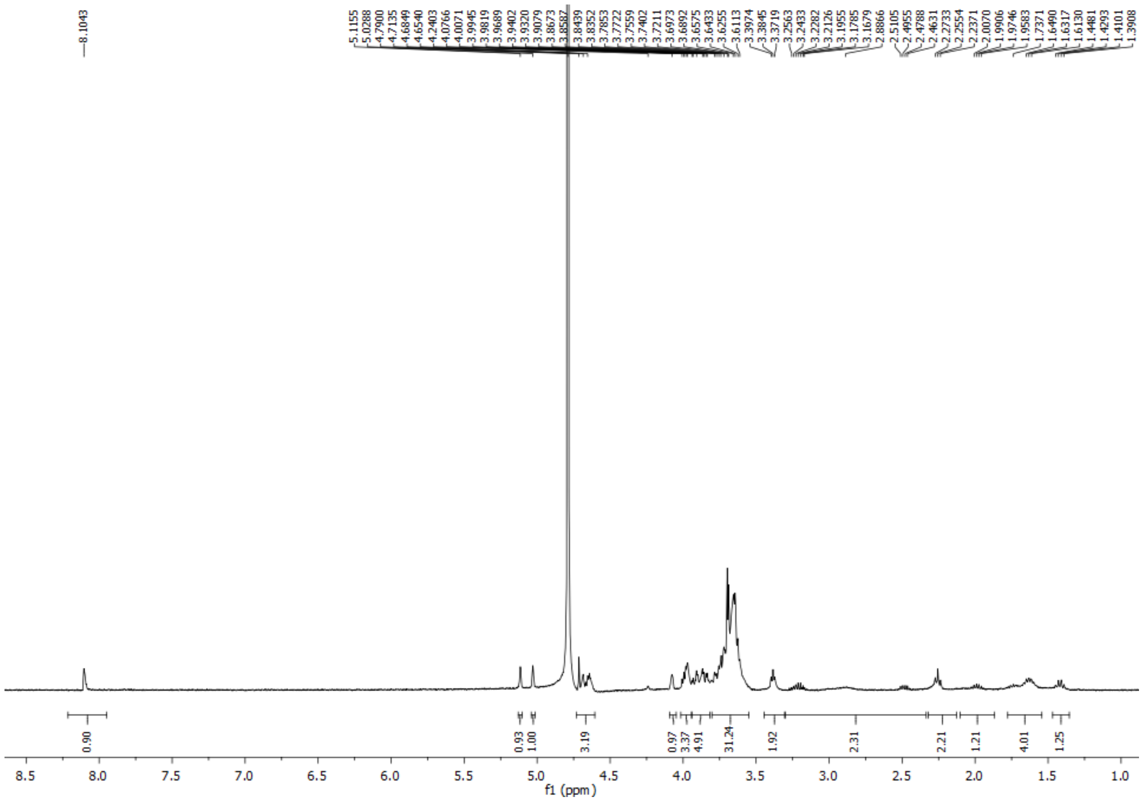

Figure S3 cont.

B- ^{13}C-NMR of LA-EG$_4$-DiMan

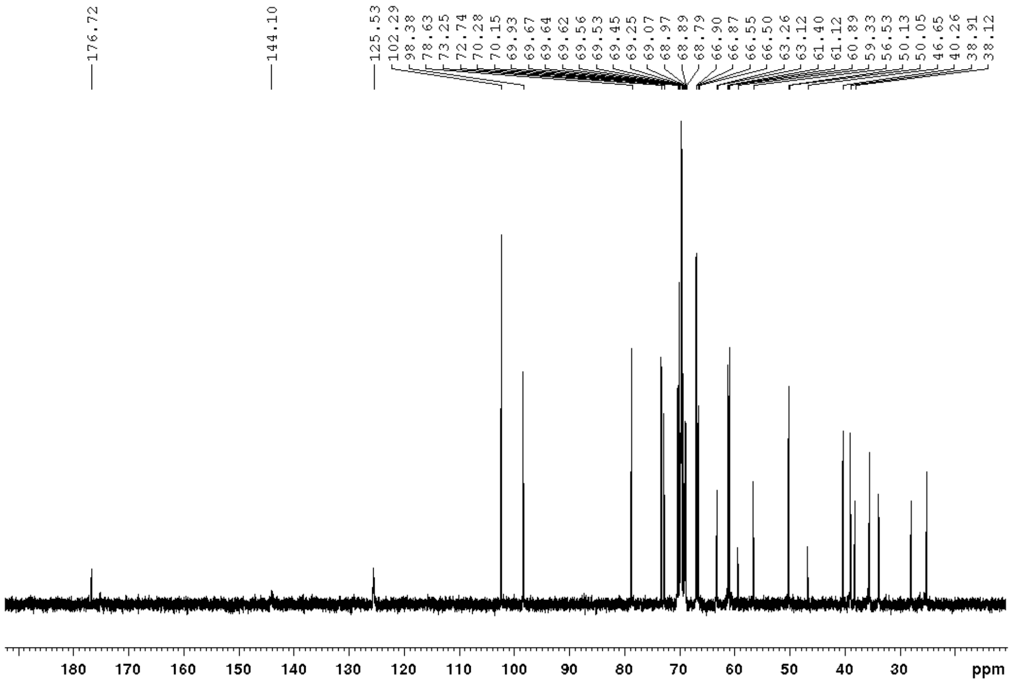

C- LC-MS of LA-EG$_4$-DiMan

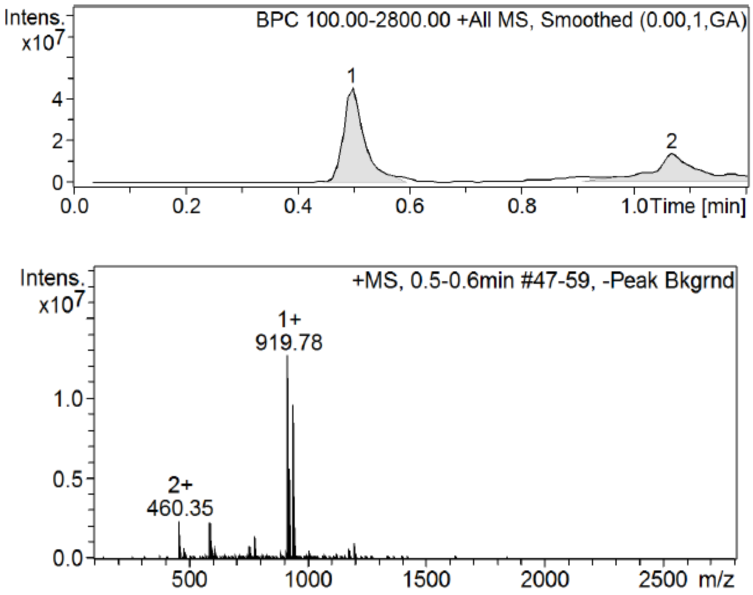

Figure S3. Spectral characterisation of LA-EG$_4$-DiMan. **(A)** ^{1}H-NMR and **(B)** ^{13}C-NMR spectrum in D_2O; and **(C)** LC-MS chromatogram and molecular ion peaks, the peak at 919.8 Da corresponds to the $(M+H)^+$ peak.

4. GNP-glycan Production and Characterisation

The conjugations of the LA-based glycan ligands onto GNPs were described in Experimental Section. Their D_h histograms were shown in Figures S4A-S4B. G13-DiMan exhibited a single D_h distribution of ~20 nm after glycan ligand conjugation, while G27-DiMan exhibited a D_h ~32 nm. Their D_h values were a few nm larger than their corresponding citrate stabled G13/G27, consistent with the G13/27 being coated with the desired LA-EG_4-DiMan ligands. Their UV-vis spectra (Figure S4C) also overlaid well with that of the parent GNPs with no significant red-shift and broadening of the SPR peaks, confirming that the formed Gx-DiMan were uniform and isolated single particle.

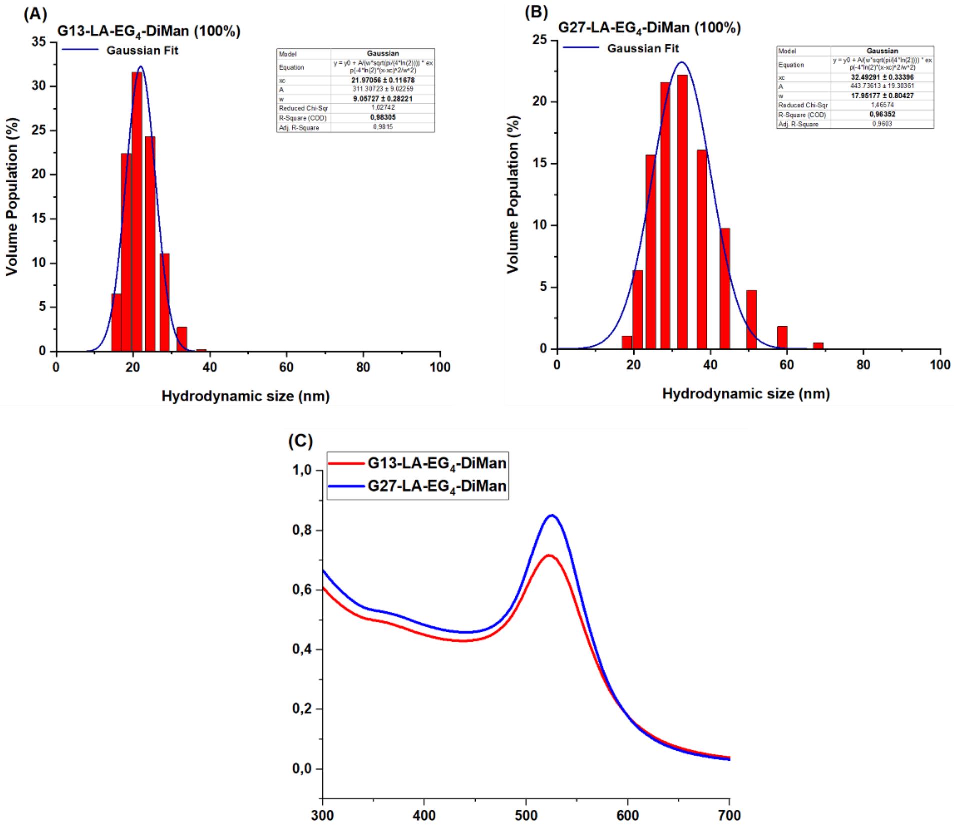

Figure S4. D_h histograms for (**A**) G13-DiMan and (**B**) G27-DiMan fitted by Gaussian function with fitting parameters shown in each graph. Data were shown in volume population. (**C**) UV-vis spectra of the G13-DiMan and G27-DiMan conjugates.

The GNP concentrations were calculated using the Beer-Lambert Law from their SPR peak absorbance at ~520 nm using an extinction coefficient of 2.32×10^8 and 2.39×10^9 $M^{-1}cm^{-1}$ for G13 and G27, respectively.

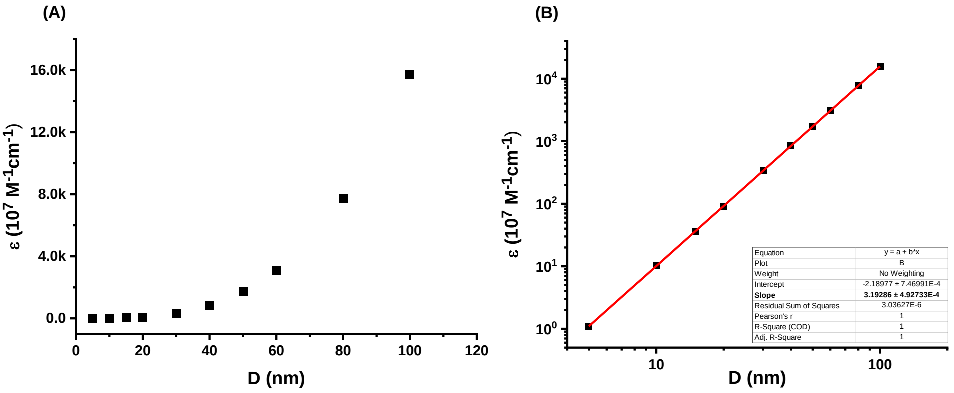

Figure S5. Plots of the GNP absorption extinction coefficient (Ɛ) as a function of diameter. The data were obtained from the Sigma-Aldrich website. (**A**) A linear-linear plot; (**B**) A log_{10}-log_{10} plot reveals a good linear relationship (R^2 = 1) with a slope of 3.19, indicating that Ɛ increases linearly with the cubic of GNP diameter ($D^{3.19}$): *i.e.* the extinction coefficient of GNP roughly scales linearly with its volume.[2, 4]

5. Determination of Glycan Valency on GNP-glycans

After GNP-glycan purification by centrifugation, the supernatants were kept and used to determine the amount of unbounded free ligands using a phenol-sulfuric acid carbohydrate quantification.[3, 5] A calibration curve was first generated using the pure LA-EG_4-DiMan ligands. 80 µL of 5% phenol solution and 400 µL of concentrated H_2SO_4 were added to a series of glycan ligand solutions in H_2O (80 µL) containing 2.0-20 µg of LA-EG_4-DiMan ligand. After the resulting mixtures immediately vortexed, they were allowed to stand at RT for 30 mins. Their absorbance at 490 nm (A_{490}) were then recorded against a black water control. The A_{490}-concentration relationship was plotted by linear function to yield a calibration curve: Y = (0.03415 ± 0.0007) x μM^{-1}. The supernatants and washing through filtrates were combined, freeze-dried, and then re-dissolved in 1.40 mL pure water. 25 µL of each solution was diluted with water to a final volume of 125 µL. This solution was then mixed with 125 µL of 5% phenol and 625 µL of H_2SO_4, and then allowed to incubate at RT for 30 mins. The absorbance of the mixture was recorded at 490 nm and the dilution factors were then corrected to calculate the total amount of unconjugated glycan ligand. The measurements were done in duplicate for each sample and the average number of LA-DiMan ligands conjugated to each GNP were calculated as 2200 ± 172 and 6290 ± 440 for G13-DiMan and G27-DiMan, respectively.[2]

5.1. Calculation of the average inter-glycan distance on GNPs

The average inter-glycan distance (d) of G13/G27-DiMan were calculated from the D_h and the glycan valency of Gx-DiMan, using the method first reported by Hill *et al.*[6] For a Gx-DiMan with a radius of r (r = 1/2 D_h) and covered with N ligand, the footprint of each ligand (k) onto Gx-DiMan hydrodynamic surface was calculated by the equation below.

$$k = \frac{4\pi r^2}{N}$$

Where *r* = 21.9/2 = 10.95 nm for G13-DiMan and 32.4/2 = 16.2 nm for G27-DiMan. The average deflection angle of each ligand on the Gx-DiMan surface (θ, in degrees) was calculated via the equation below.

$$\theta = \frac{2 \times 180\, x \sqrt{\frac{k}{\pi}}}{r\pi}$$

The estimated θ for G13-DiMan and G27-DiMan (N = 2200 and 6290) were 4.88° and 2.89°, respectively. Using these data, the inter-glycan distance on the Gx-DiMan surface (X) was then calculated *via* the following equation and the results were summarised in Table S1 below.

$$X = 2\, r\, \sin\left(\frac{\theta}{2}\right)$$

Table S1. Summary of the chemical and physical parameters of the G13/27-DiMan conjugates.

GNP-glycan	Glycan valency	D_h (nm)	Glycan footprint on GNP surface (nm^2)	Inter-glycan spacing (d) in nm
**G13-EG_4-DiMan**	2200 ± 170	21.9 nm	0.684	~0.93 nm
**G27-EG_4-DiMan**	6290 ± 440	32.4 nm	0.524	~0.80 nm

6. Protein Production and Characterisation

6.1. Wild-Type DC-SIGN and DC-SIGNR Production and Characterisation

Wild-type DC-SIGN/R were expressed from *E. coli.* and purified by sepharose-mannose affinity columns as described previously. Their concentrations were determined from their absorbances at 280 nm using an extinction coefficient of 70 400 and 60 890 M^{-1} cm^{-1} per DC-SIGN and DC-SIGNR monomer, respectively.[3, 5] Their identities were confirmed by high resolution mass spectrometry (HRMS, Figure S6). The calculated MWs are 39197.22 and 37478.99 for DC-SIGN and DC-SIGNR, respectively, found 39201.61 and 37470.40.

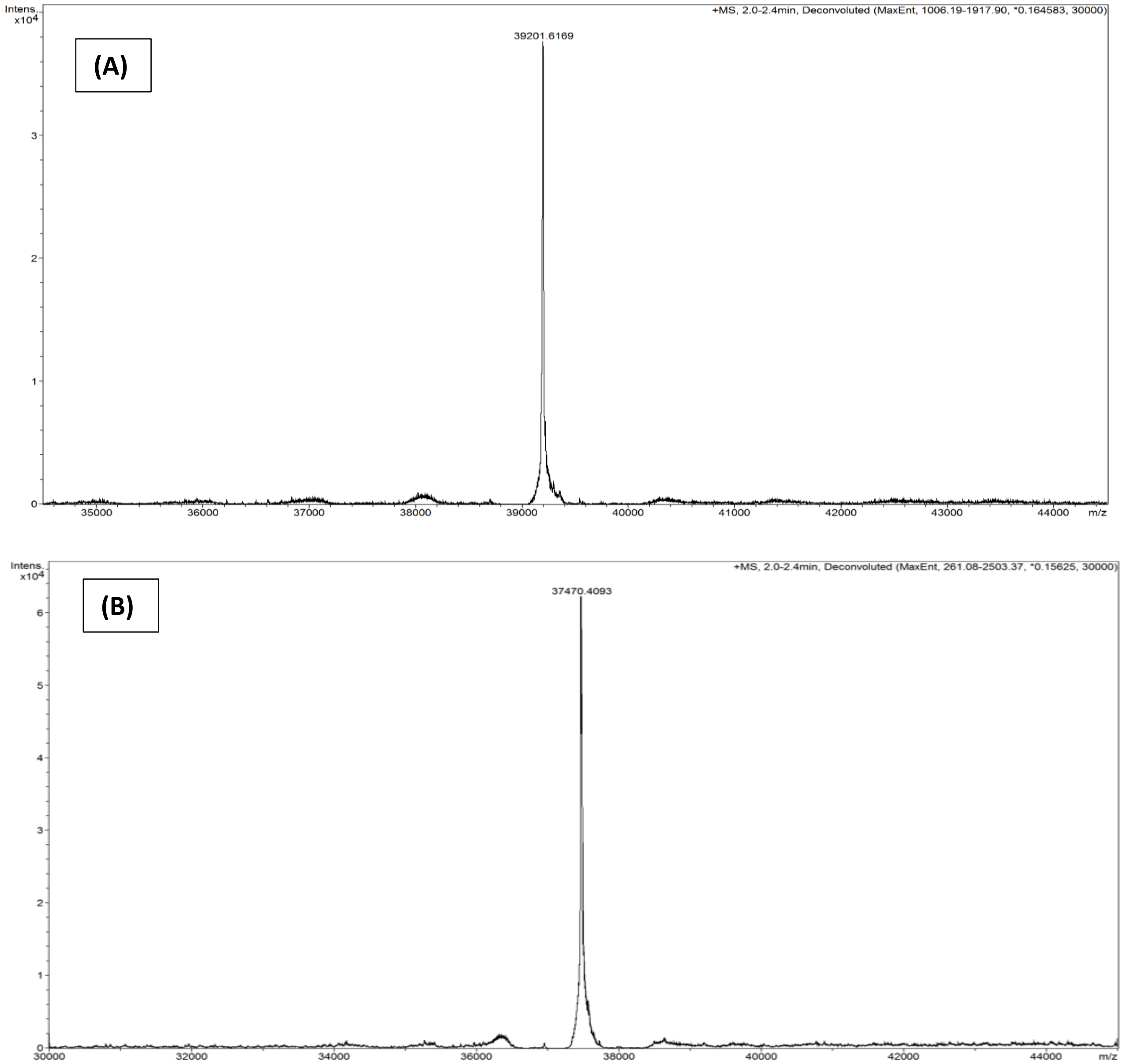

Figure S6. HR-MS spectra of (A) wild-type DC-SIGN and (B) DC-SIGNR.

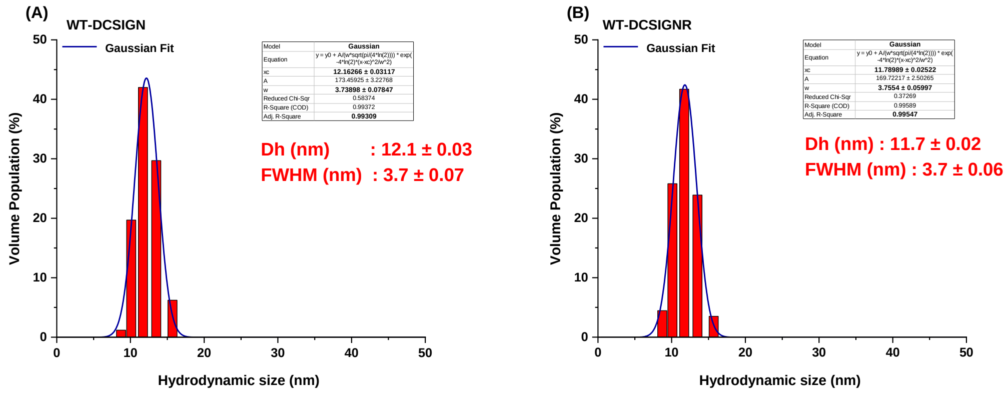

Figure S7. D_h (volume population) distribution histograms for (**A**) wild-type DC-SIGN; (**B**) wild-type DC-SIGNR. They were fitted by Gaussian function with fitting parameters and data were shown in volume population.

6.2. DC-SIGN Q274C-ATTO643 and DC-SIGNR R287C-ATTO643 Production and Characterisation

The mutant proteins DC-SIGN Q274C and DC-SIGNR R287C were produced as described previously.[3, 5] They were concentrated using 10 kDa MWCO spin filters in a labelling buffer (20 mM HEPES, 150 mM NaCl, 25 mM $CaCl_2$, pH 7.2) and then Atto-643 (10 mg/mL in dry DMSO) was added at a molar ratio of 2:1 to the protein monomer. The mixture was vortexed, wrapped with aluminium foil and gently stirred at RT for 2 hours and then kept at +4 ^{0}C in a fridge overnight. After that, the proteins were purified by mannose-sepharose affinity column and characterised by HRMS as shown in Figure S8 below.

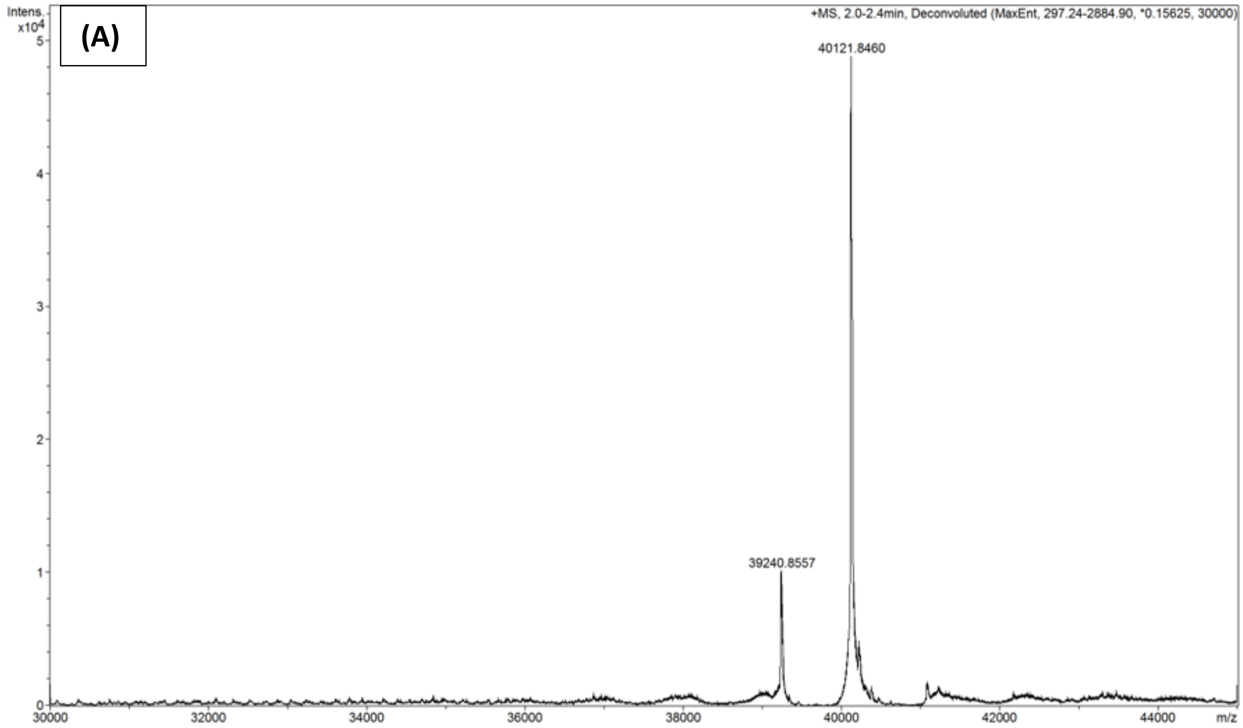

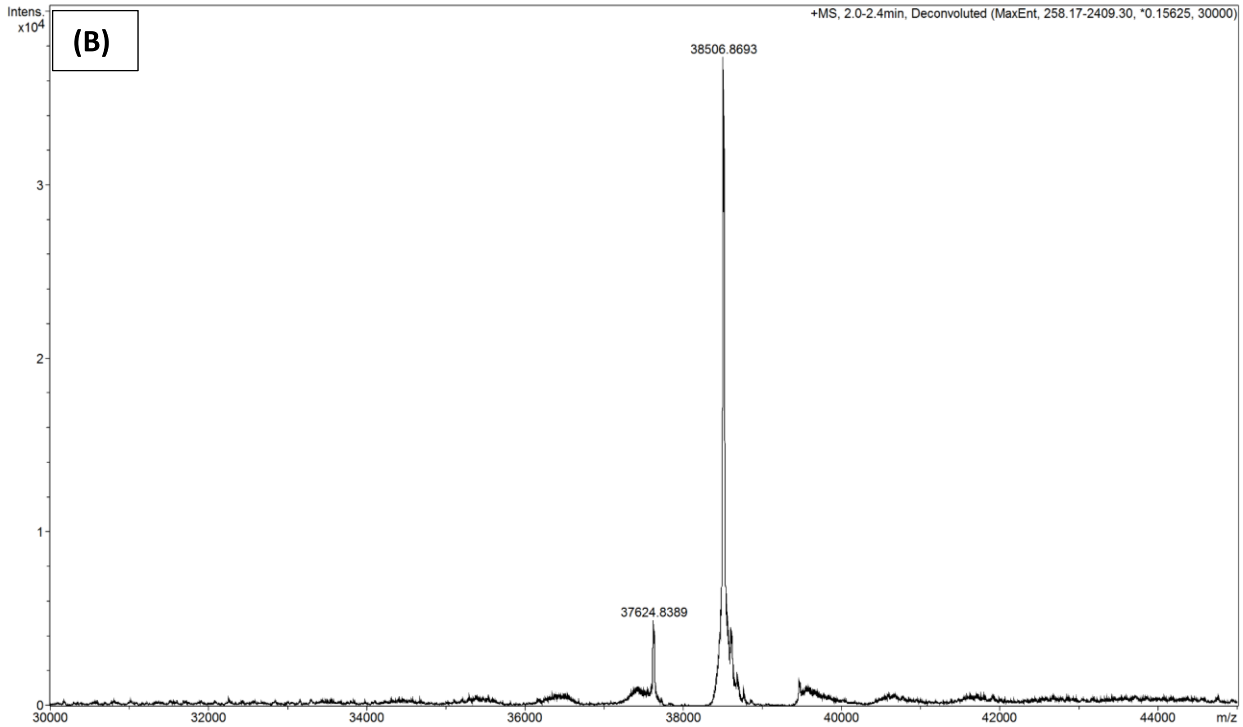

Figure S8. HR-MS spectra of (**A**) DC-SIGN-Atto 643 and (**B**) DC-SIGNR-Atto 643. An increase of MW of ~882 Da, corresponding to Atto-643 dye, was observed form both proteins. The dye labelling efficiency (per protein monomer) was estimated as ~82% and ~90% for DC-SIGN and DC-SIGNR, respectively, based on the labeled peak area abundance to the total proteins.

7. Fluorescence Spectra for DC-SIGN, DC-SIGNR, and Atto-643 dye at Different pHs

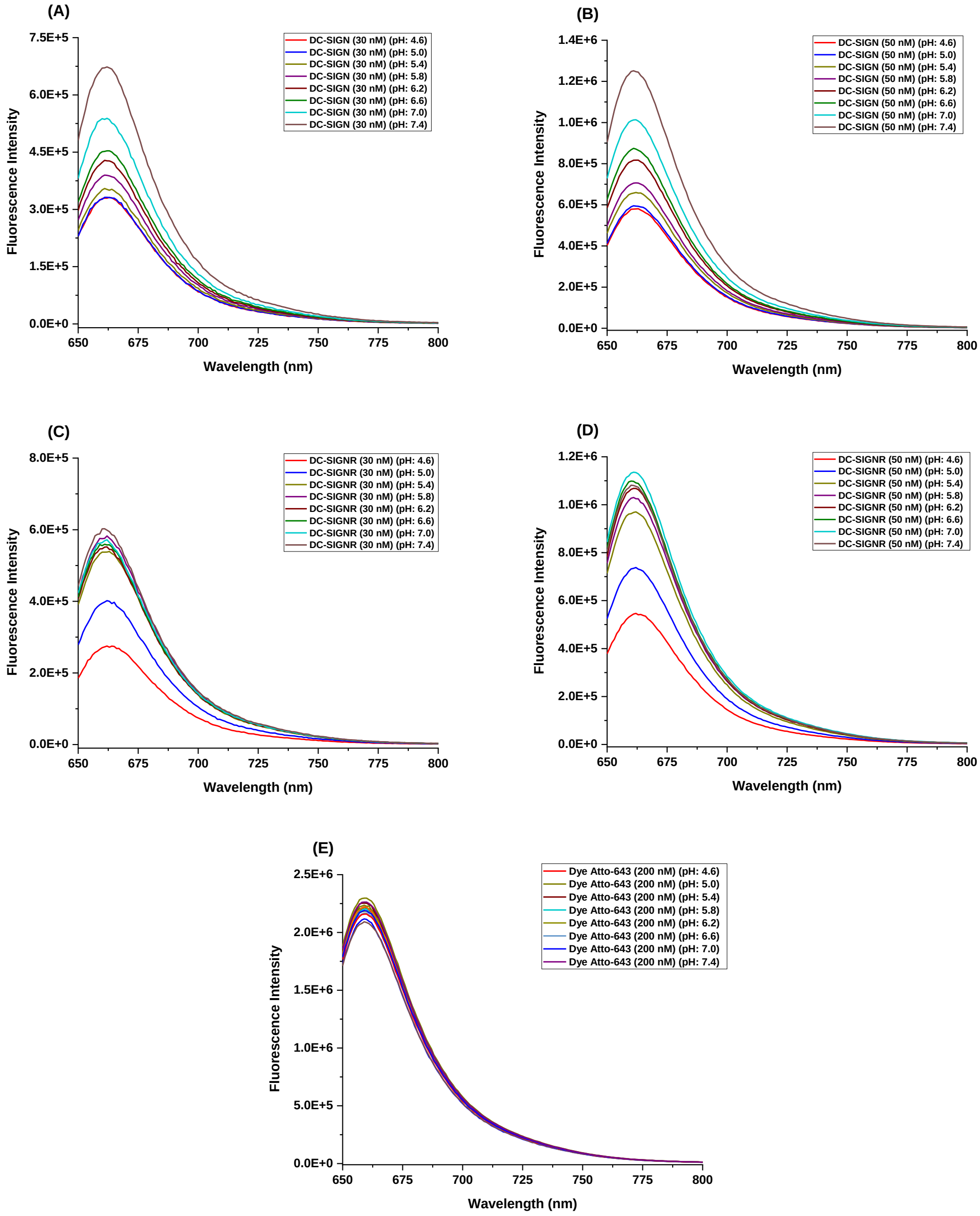

Figure S9. The fluorescence spectra for (**A**) DC-SIGN at 30 nM, (**B**) DC-SIGN at 50 nM, (**C**) DC-SIGNR at 30 nM, (**D**) DC-SIGNR at 50 nM, and (**E**) Atto-643 dye at different pH points over the range from 4.6 to 7.4.

8. Fluorescence Spectra for G13/27-DiMan+DC-SIGN/R at Different pHs.

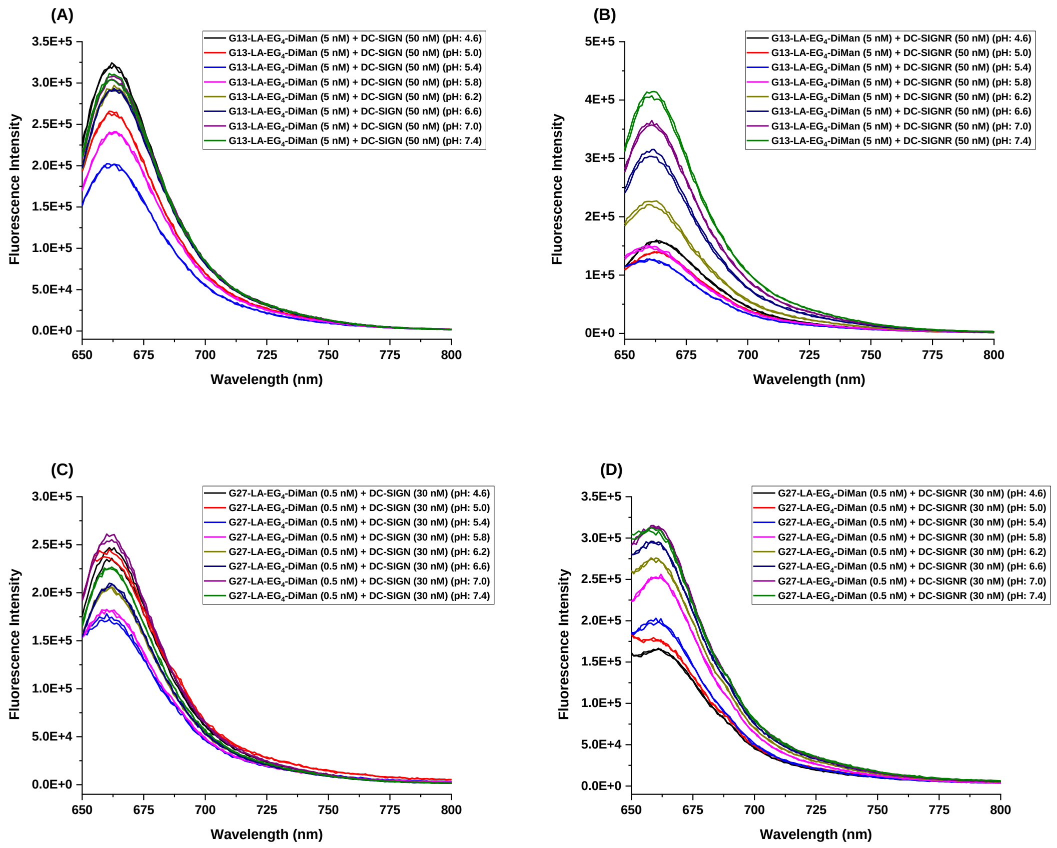

Figure S10. The fluorescence spectra for (A) G13-EG_4-DiMan + DC-SIGN, (B) G13-EG_4-DiMan + DC-SIGNR, (C) G27-EG_4-DiMan + DC-SIGN, (D) G27-EG_4-DiMan + DC-SIGNR at different pH points over the range from 4.6 to 7.4.

9. Hydrodynamic Size Measurements of GNP-DiMan+DC-SIGN/R at Different pHs

9.1 Pure G13-EG_4-DiMan or DC-SIGN alone

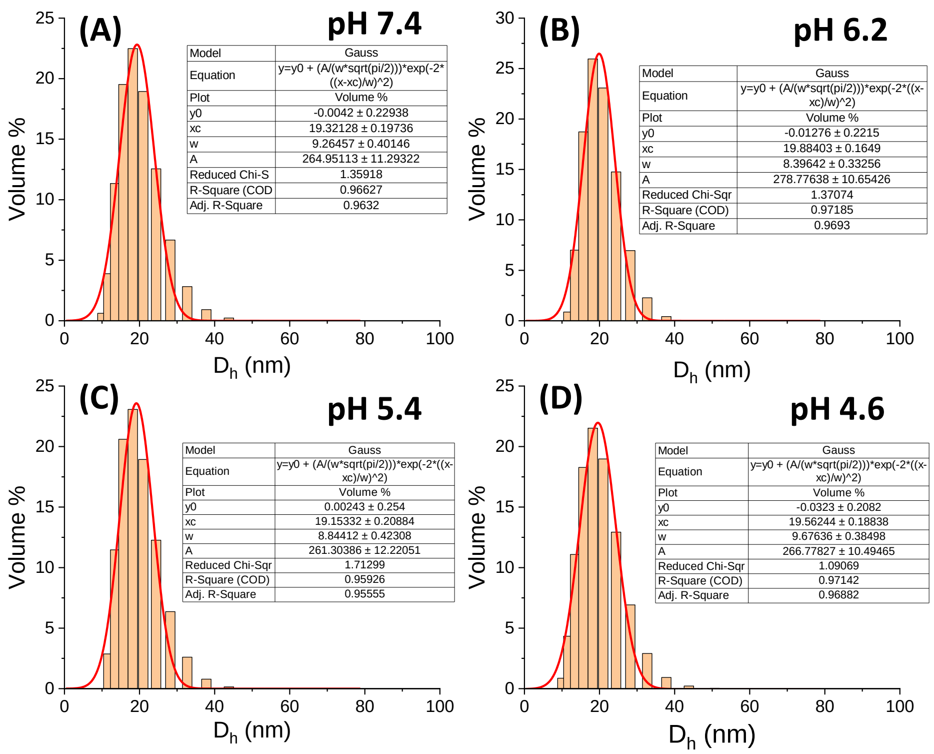

Figure S11. D_h distribution histograms (volume population) of G13-EG4-DiMan (5 nM) in a MES buffer under different pHs: (A) 7.4; (B) 6.2, (C) 5.4 and (D) 4.6.

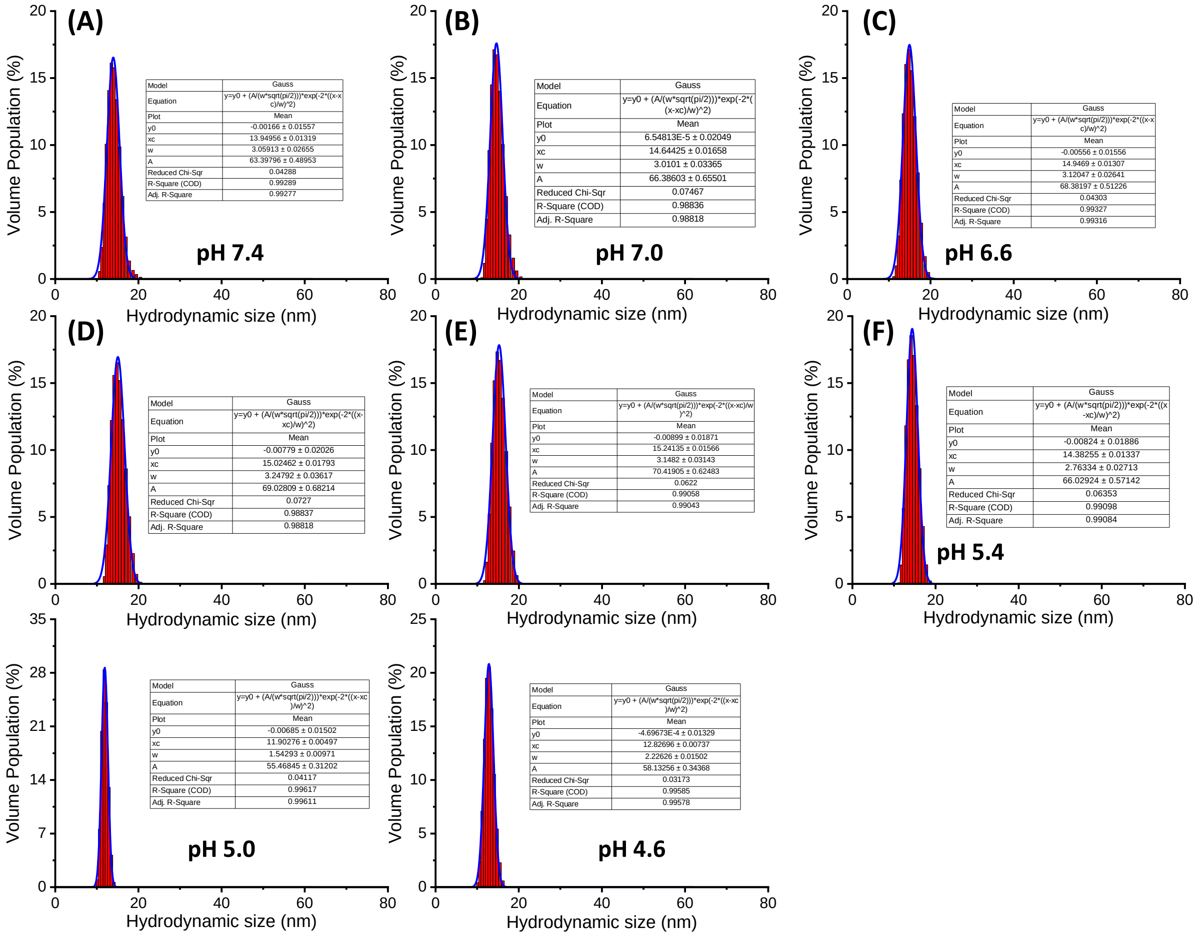

Figure S12: D_h distribution histograms (volume population) of DC-SIGN (50 nM) in a MES buffer under different pHs: (A) 7.4; (B) 7.0; (C) 6.6; (D) 6.2, (E) 5.8; (F) 5.4; (G) 5.0 and (H) 4.6.

9.2 G13-EG$_4$-DiMan + DC-SIGN

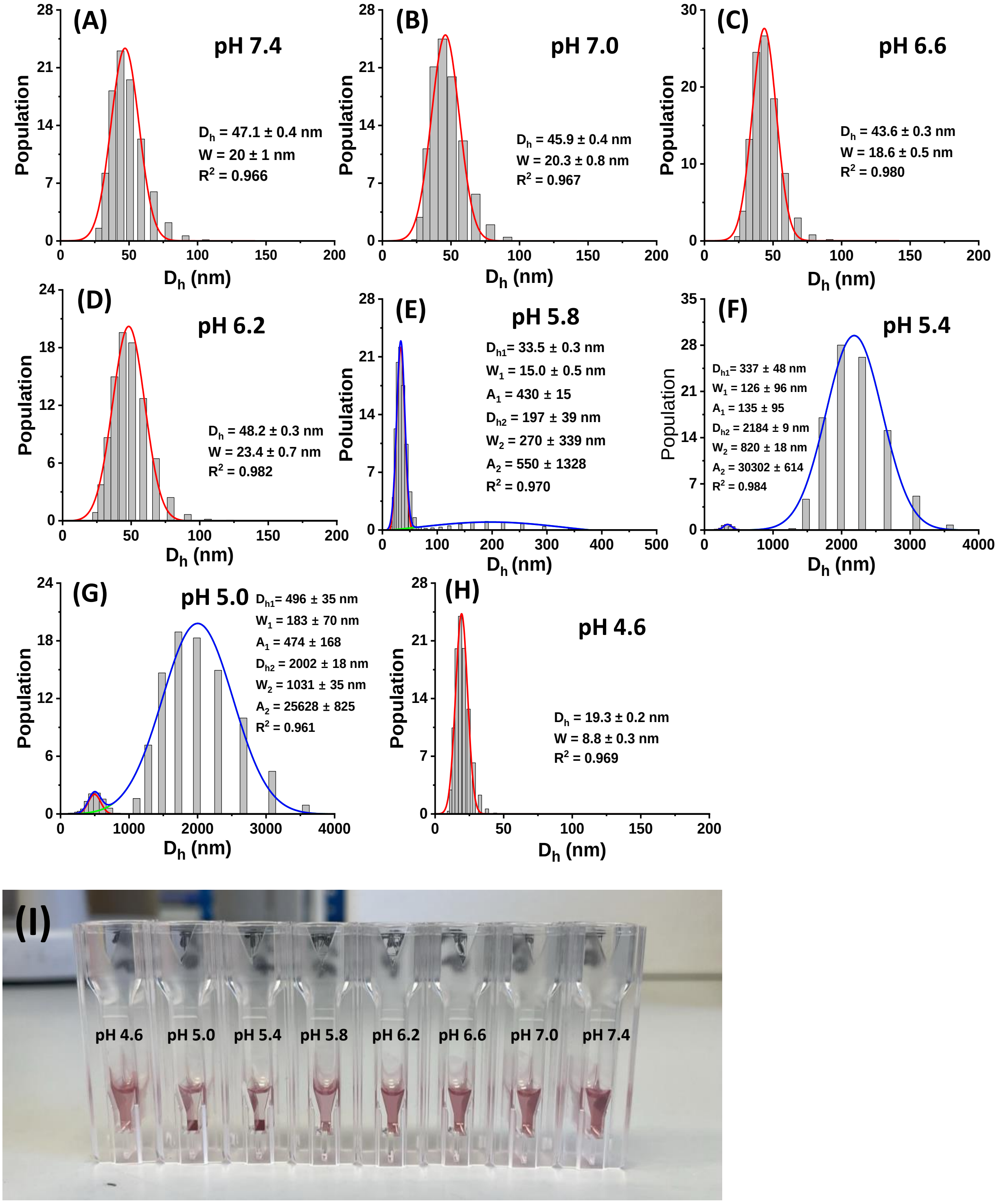

Figure S13. (A-H) The DLS histograms of G13-EG$_4$-DiMan (5 nM) + DC-SIGN (50 nM) samples at (A) pH 7.4, (B) pH 7.0, (C) pH 6.6, (D) pH 6.2, (E) pH 5.8, (F) pH 5.4, (G) pH 5.0, and (H) pH 4.6. (**I**) An optical photograph of the G13-EG$_4$-DiMan + DC-SIGN samples used in DLS measurement after standing overnight. Samples at pHs of 4.6, and 6.2 to 7.4 remained red and showed no significant changes; while that at pH 5.8 showed reduced colour and those at pH 5.0 and 5.4 have precipitated out of the solution.

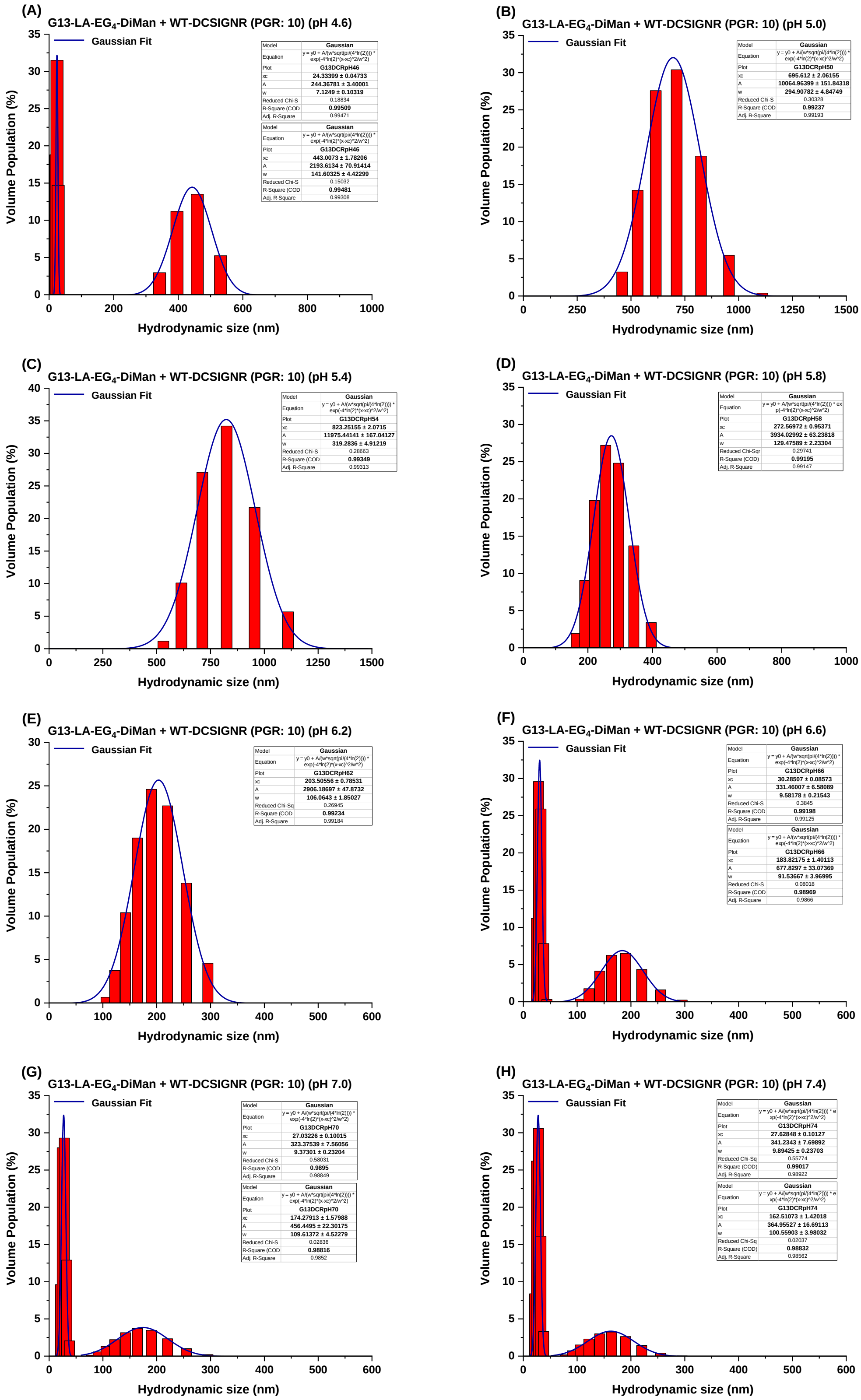

Figure S14. The DLS histograms of G13-EG$_4$-DiMan (5 nM) + DC-SIGNR (50 nM) at (A) pH 4.6, (B) pH 5.0, (C) pH 5.4, (D) pH 5.8, (E) pH 6.2, (F) pH 6.6, (G) pH 7.0, (H) pH 7.4.

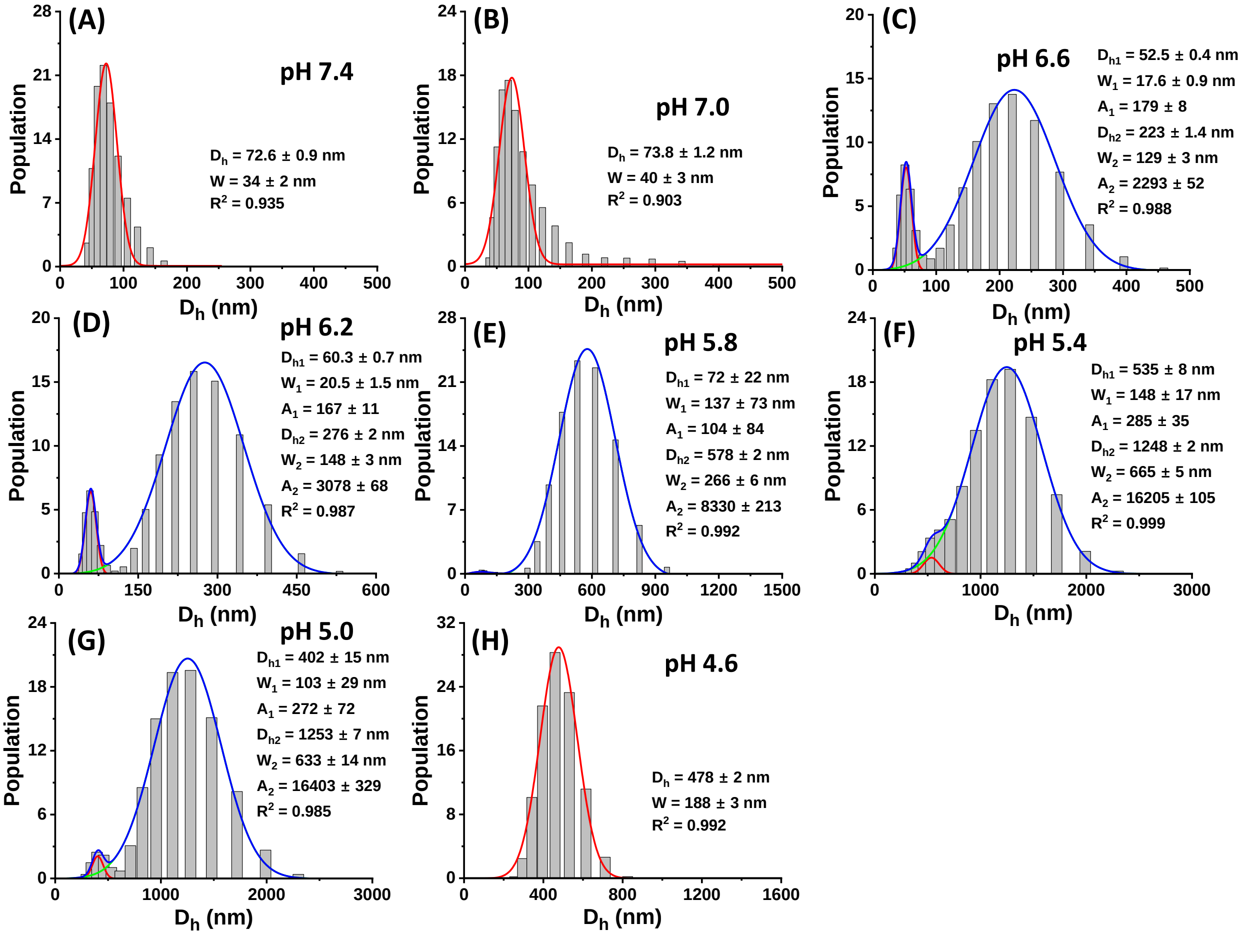

Figure S15. The representative DLS histograms (volume population) of G27-EG$_4$-DiMan (0.5 nM) + DC-SIGN (30 nM) samples at (A) pH 7.4, (B) pH 7.0, (C) pH 6.6, (D) pH 6.2, (E) pH 5.8, (F) pH 5.4, (G) pH 5.0, and (H) pH 4.6.

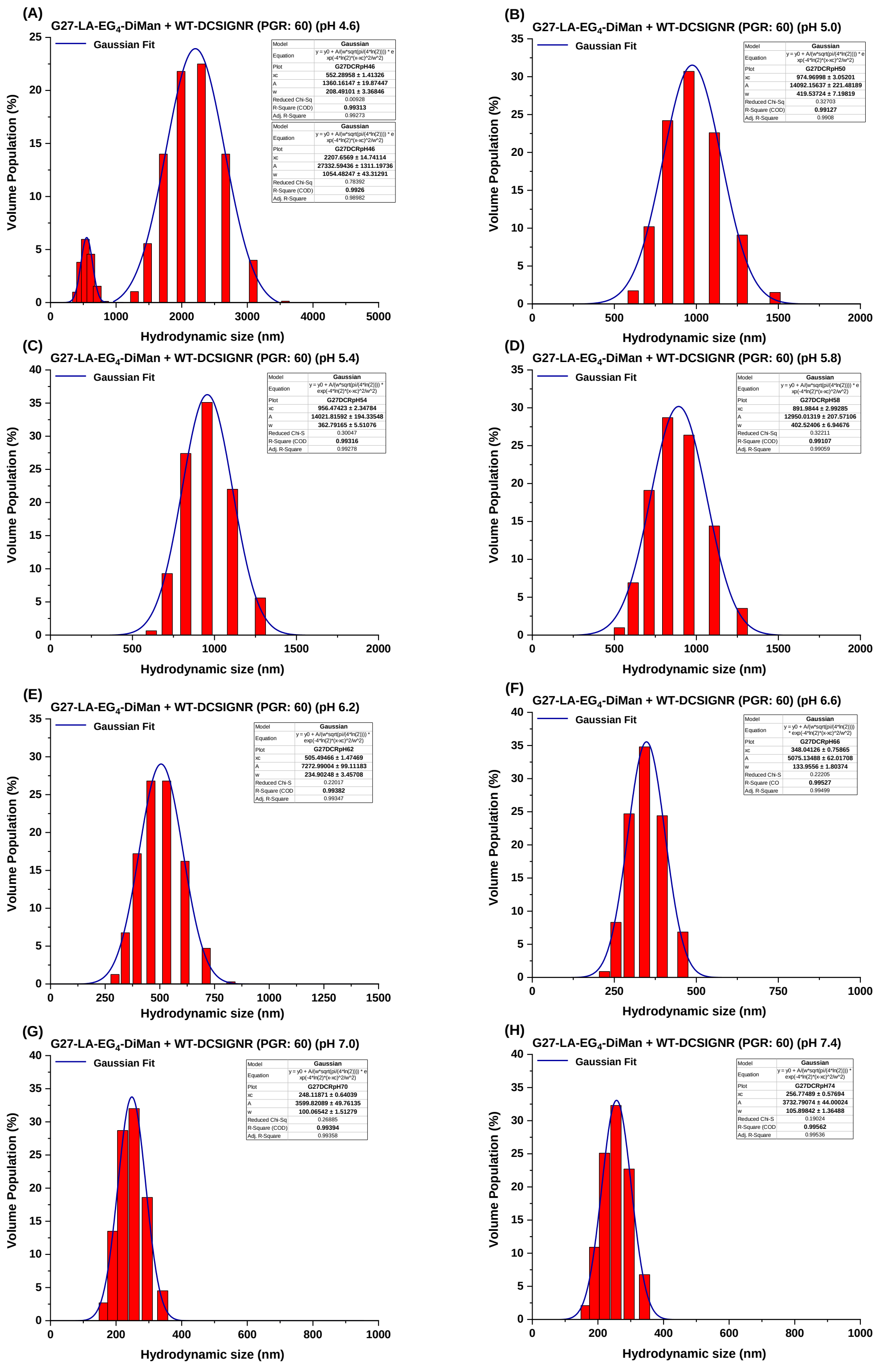

Figure S16. The DLS histograms of G27-EG$_4$-DiMan (0.5 nM) + DC-SIGNR (30 nM) at (A) pH 4.6, (B) pH 5.0, (C) pH 5.4, (D) pH 5.8, (E) pH 6.2, (F) pH 6.6, (G) pH 7.0, (H) pH 7.4.

10. pH-Switching Studies

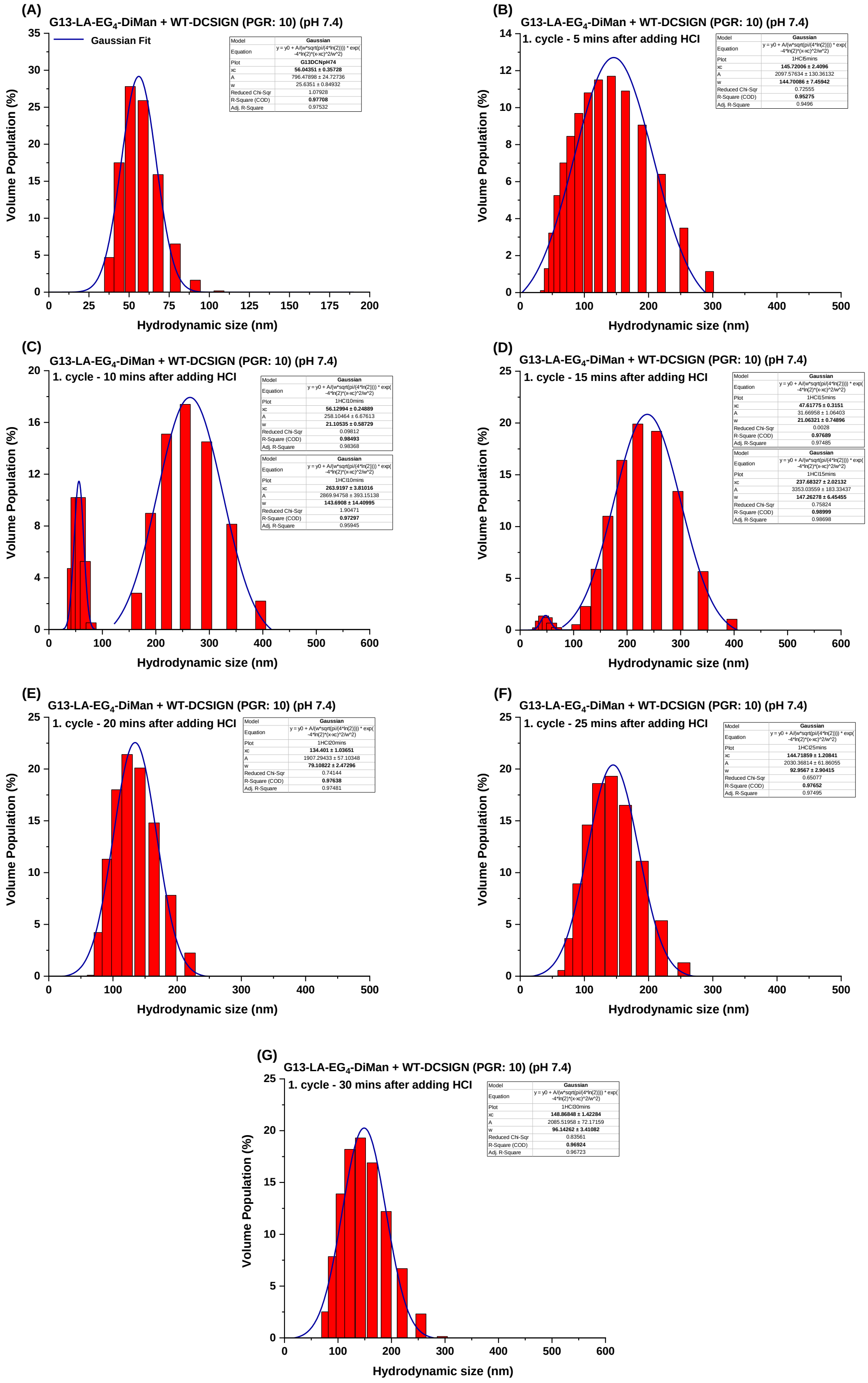

Figure S17. D_h (volume population) histograms for a G13-EG$_4$-DiMan (5 nM) + DC-SIGN (50 nM) sample starting at pH 7.4 (A) after adding HCl to switch pH from 7.4 to 5.0 (cycle 1) over time: (B) 5 mins, (C) 10 mins; (D) 15 mins; (E) 20 mins; (F) 25 mins; (G) 30 mins.

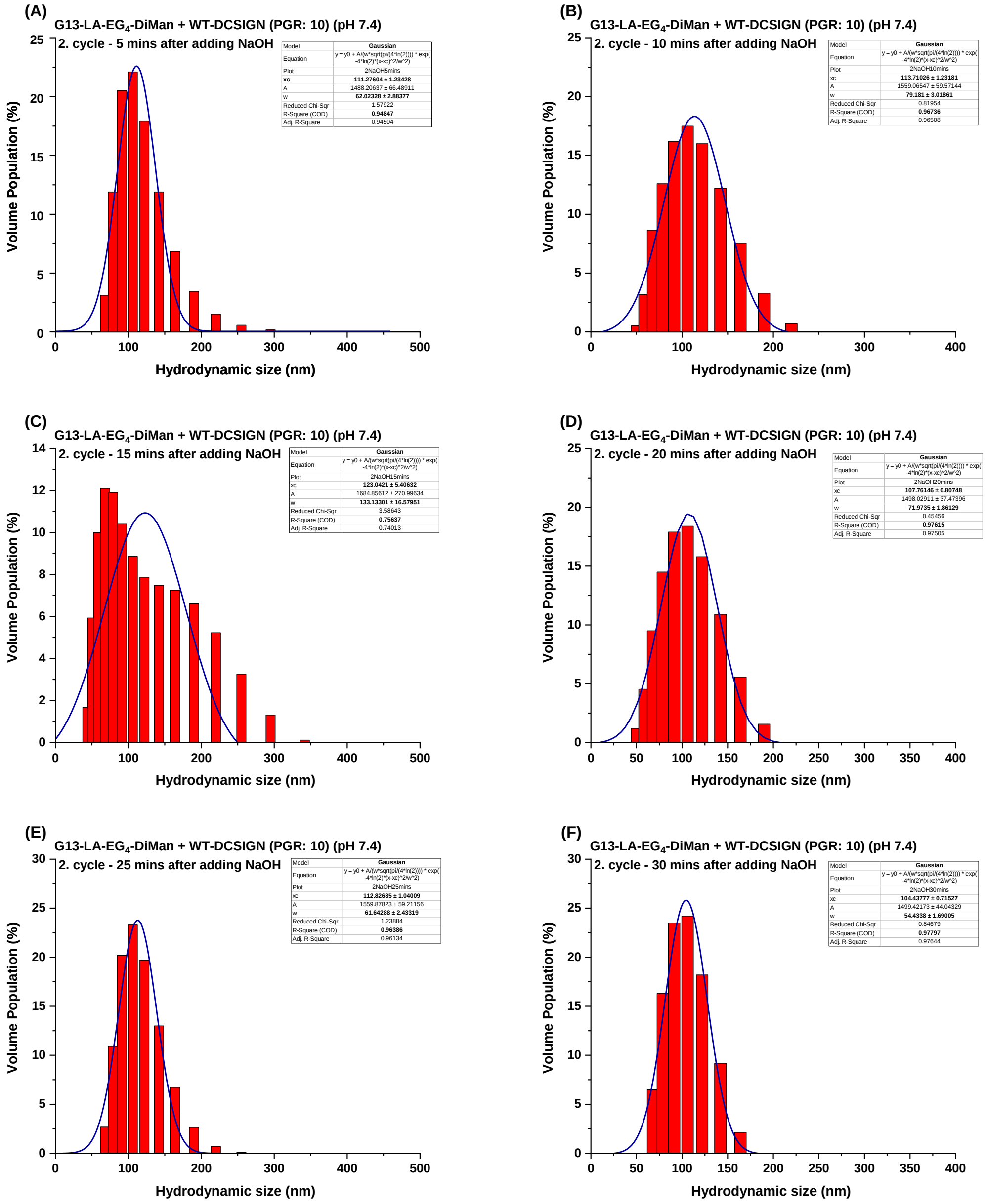

Figure S18. D_h histograms of G13-EG$_4$-DiMan (5 nM) + DC-SIGN (50 nM) sample as pH is switched from 5.0 to 7.4 by adding NaOH (cycle 2) over time: (A) 5 mins, (B) 10 mins; (C) 15 mins; (D) 20 mins; (E) 25 mins; (F) 30 mins.

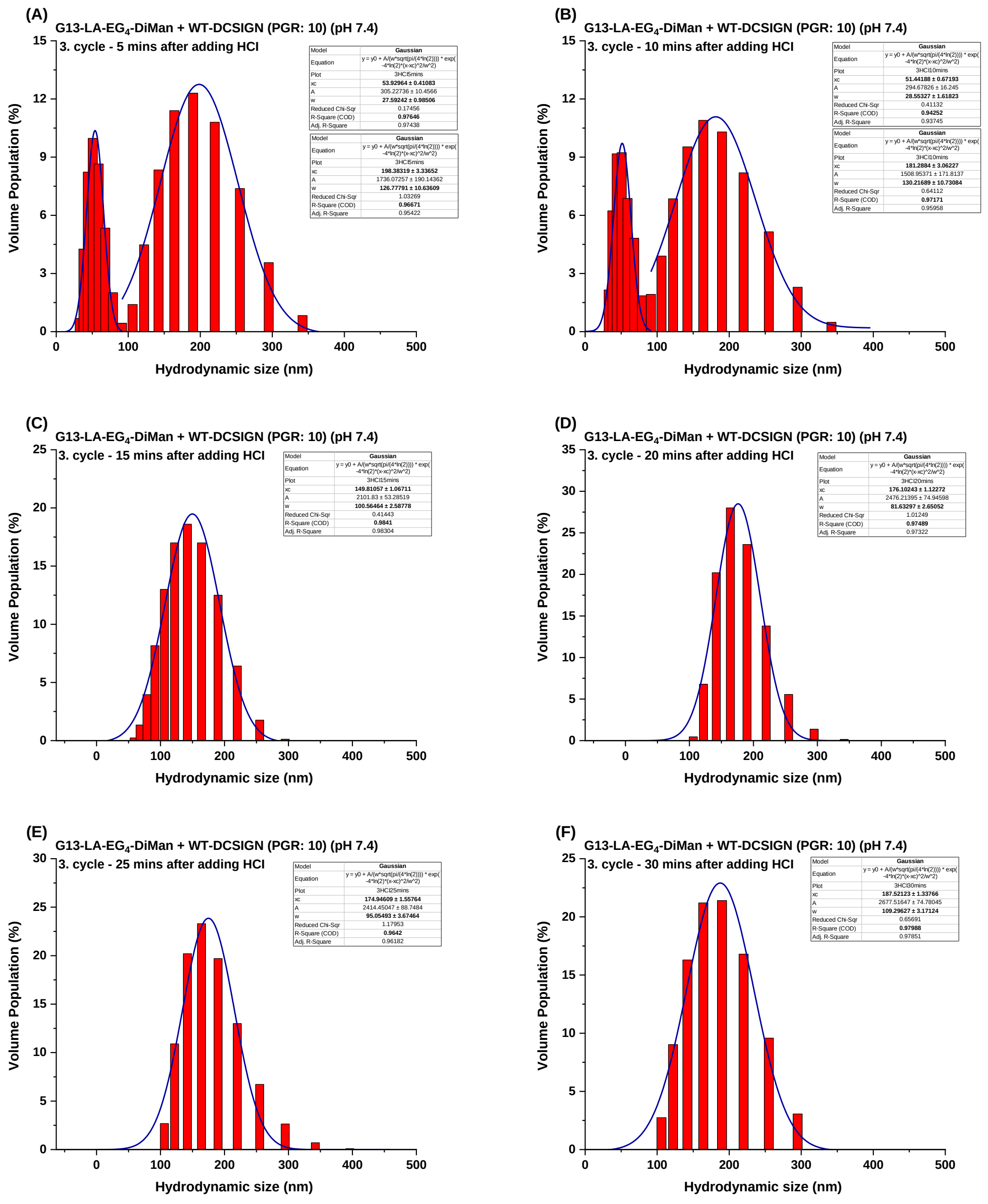

Figure S19. D_h histograms of G13-EG$_4$-DiMan (5 nM) + DC-SIGN (50 nM) sample as pH is switched from 7.4 to 5.0 by adding HCl (cycle 3) over time: (A) 5 mins, (B) 10 mins; (C) 15 mins; (D) 20 mins; (E) 25 mins; (F) 30 mins.

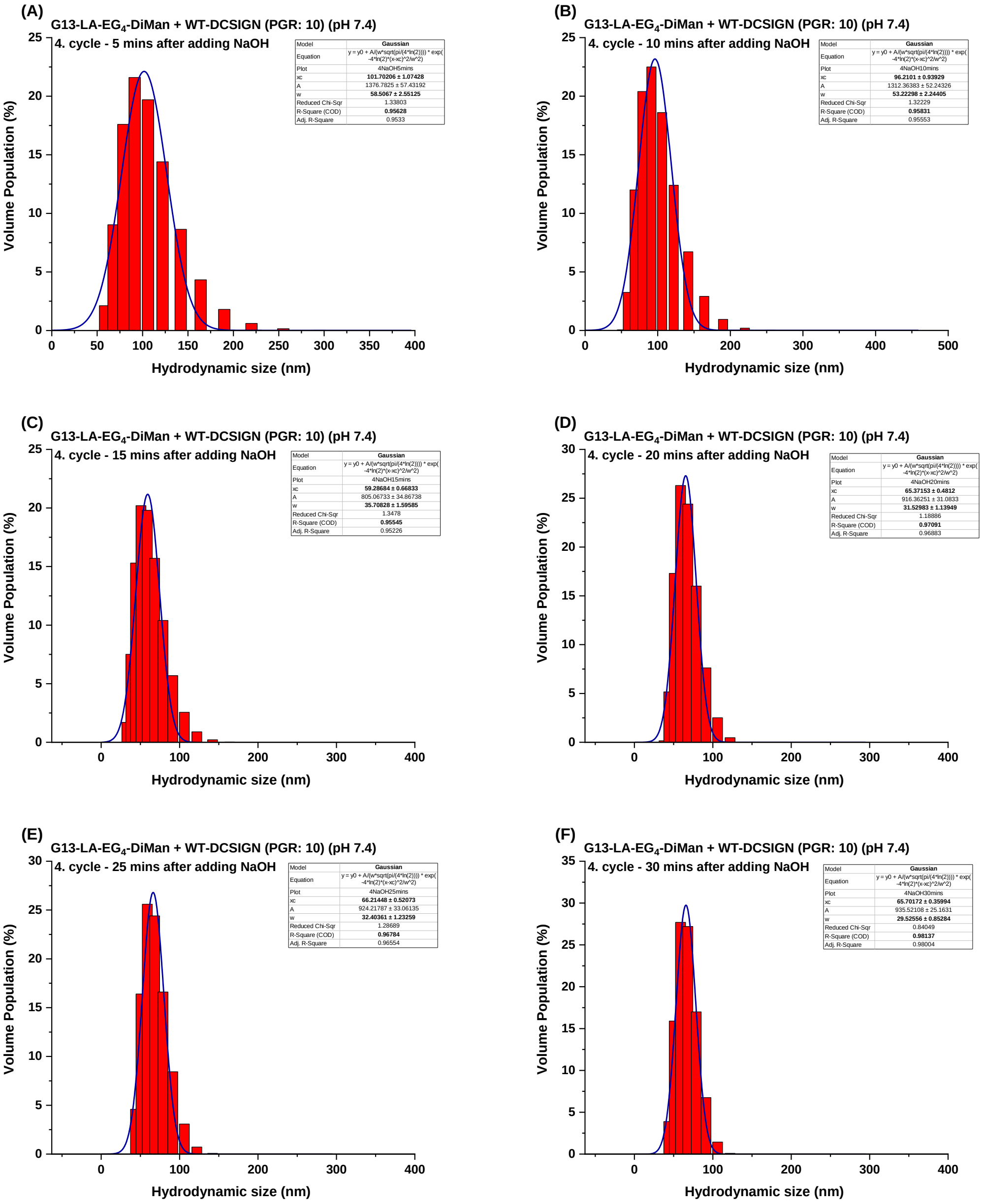

Figure S20. D_h histograms of G13-EG$_4$-DiMan (5 nM) + DC-SIGN (50 nM) sample as pH is switched from 5.0 to 7.4 by adding NaOH (cycle 4) over time: (A) 5 mins, (B) 10 mins; (C) 15 mins; (D) 20 mins; (E) 25 mins; (F) 30 mins.

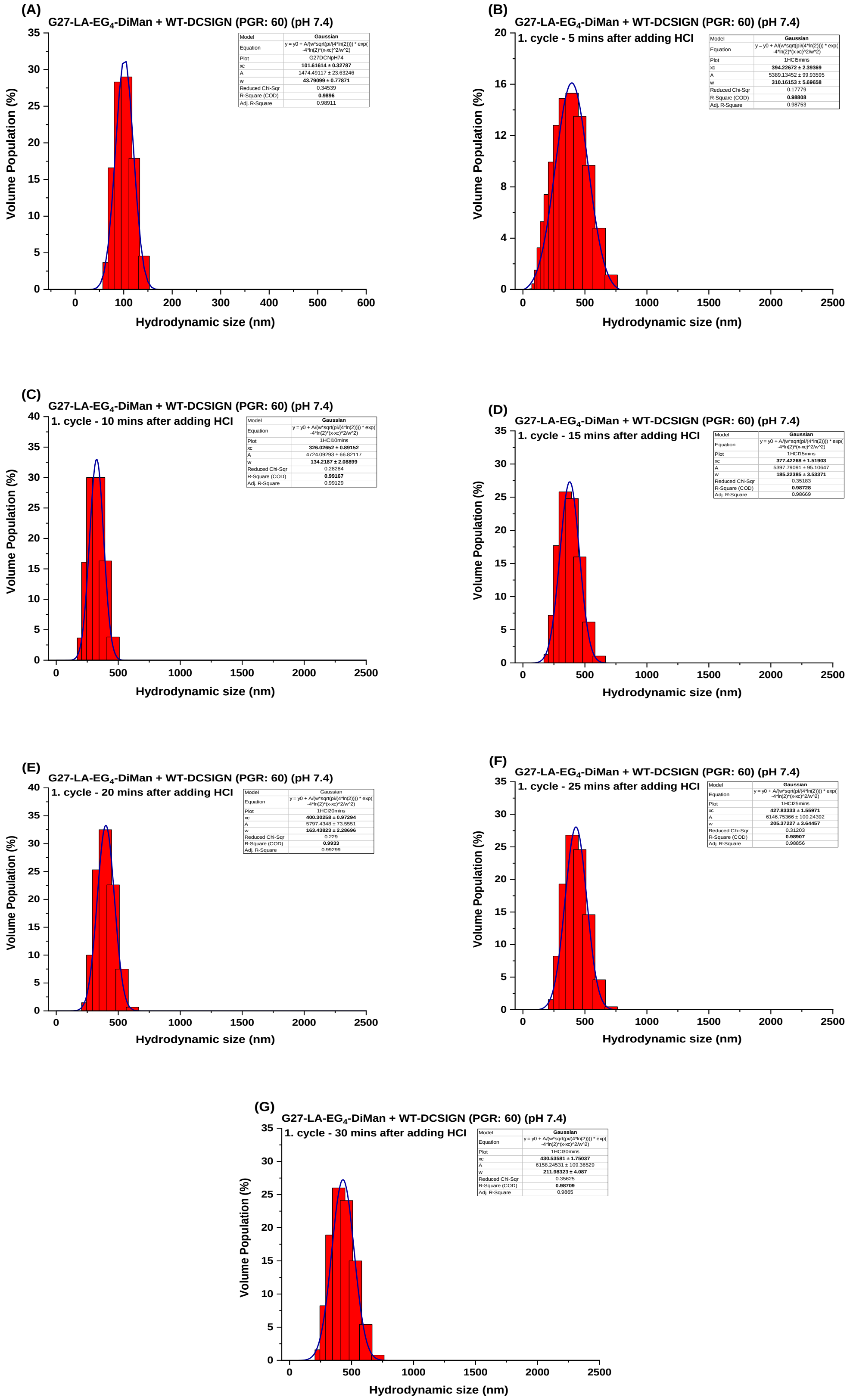

Figure S21. D_h histograms of G27-EG$_4$-DiMan (0.5 nM) + DC-SIGN (30 nM) sample starting at pH 7.4 (A) as pH is switched to 5.0 by adding HCl (cycle 1) over time: (B) 5 mins, (C) 10 mins; (D) 15 mins; (E) 20 mins; (F) 25 mins; (G) 30 mins.

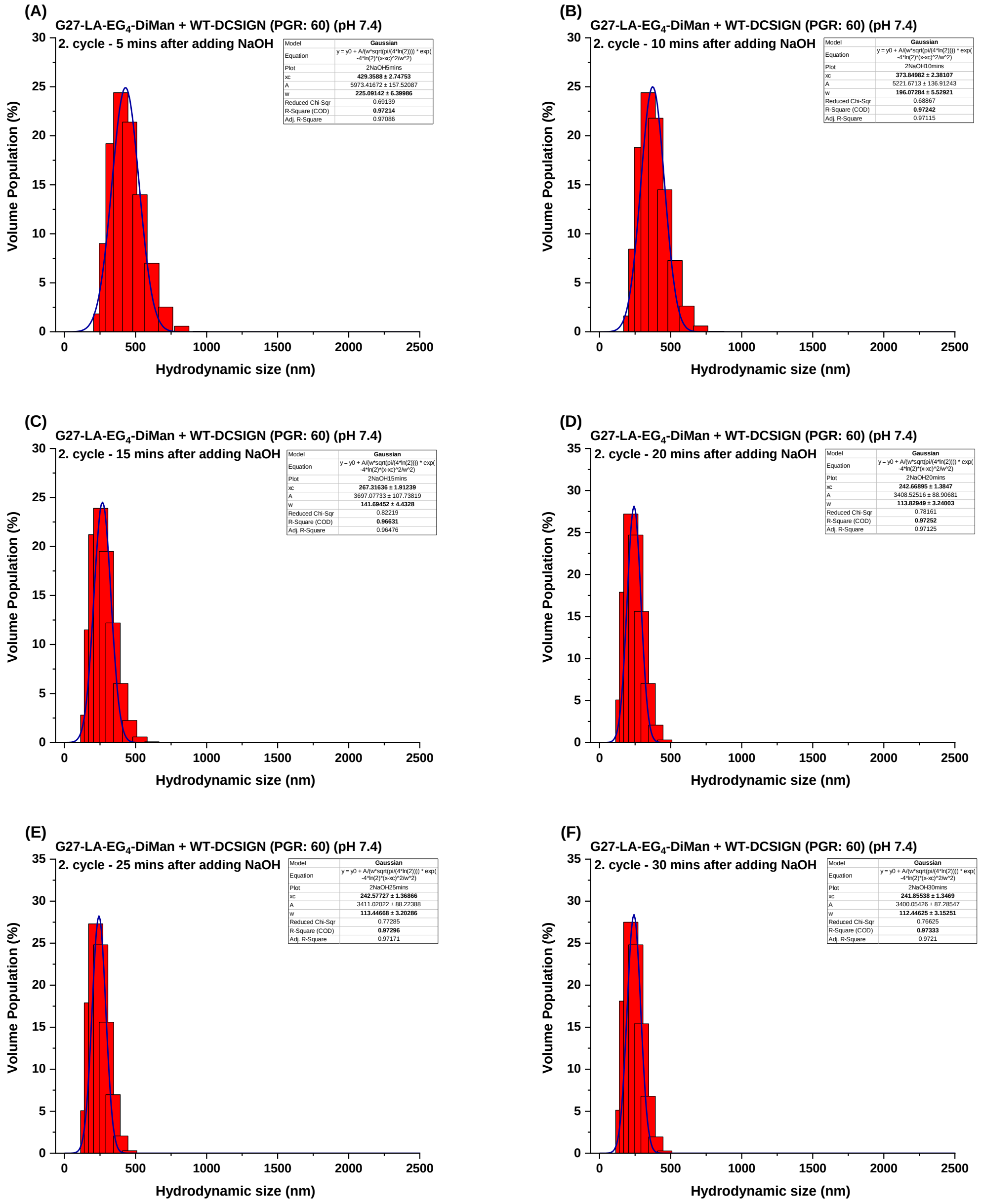

Figure S22. D_h histograms of G27-EG$_4$-DiMan (0.5 nM) + DC-SIGN (30 nM) sample as pH is switched from 5.0 to 7.4 by adding NaOH (cycle 2) over time: (A) 5 mins, (B) 10 mins; (C) 15 mins; (D) 20 mins; (E) 25 mins; (F) 30 mins.

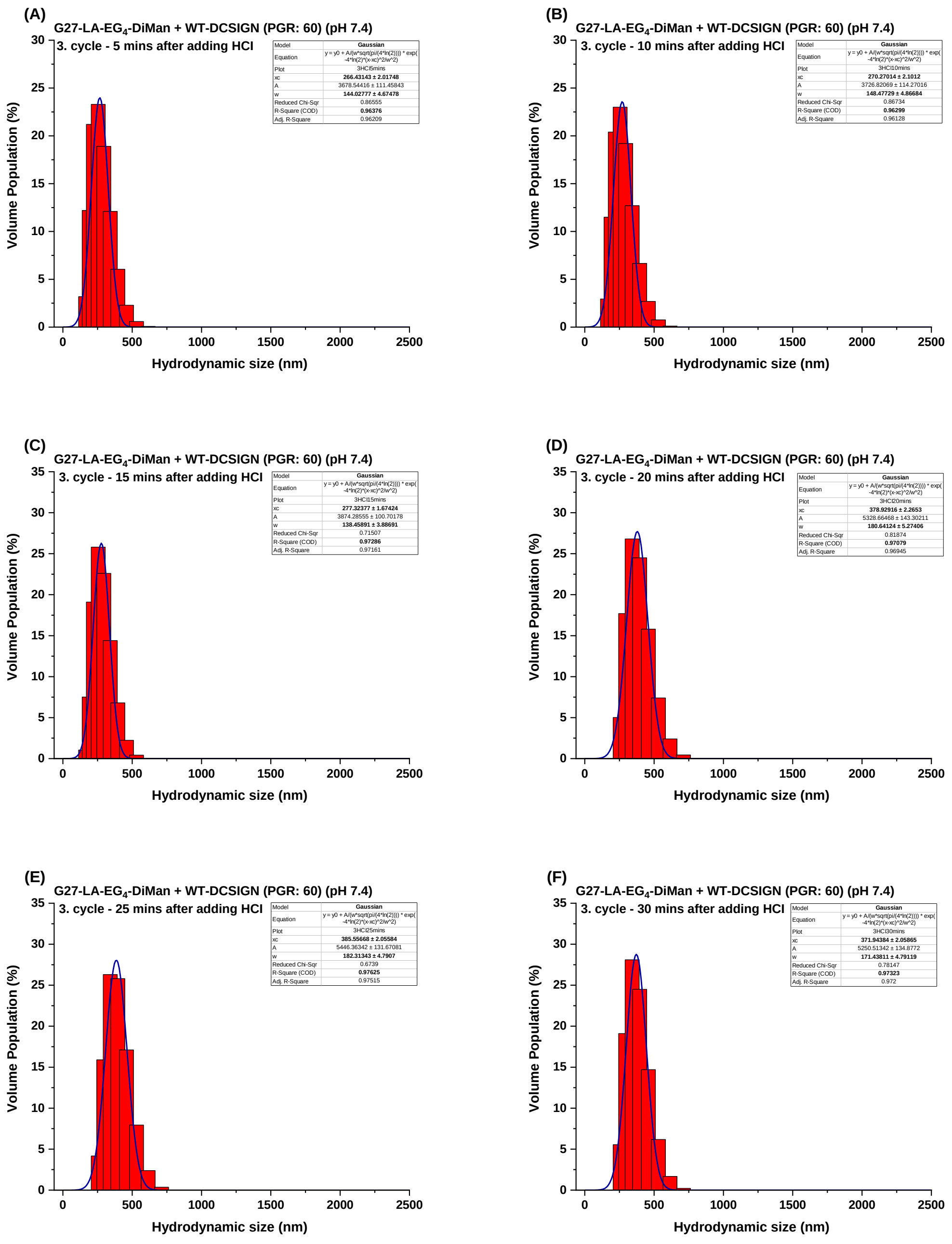

Figure S23. D_h histograms of G27-EG$_4$-DiMan (0.5 nM) + DC-SIGN (30 nM) sample as pH is switched from 7.4 to 5.0 by adding HCl (cycle 3) over time: (A) 5 mins, (B) 10 mins; (C) 15 mins; (D) 20 mins; (E) 25 mins; (F) 30 mins.

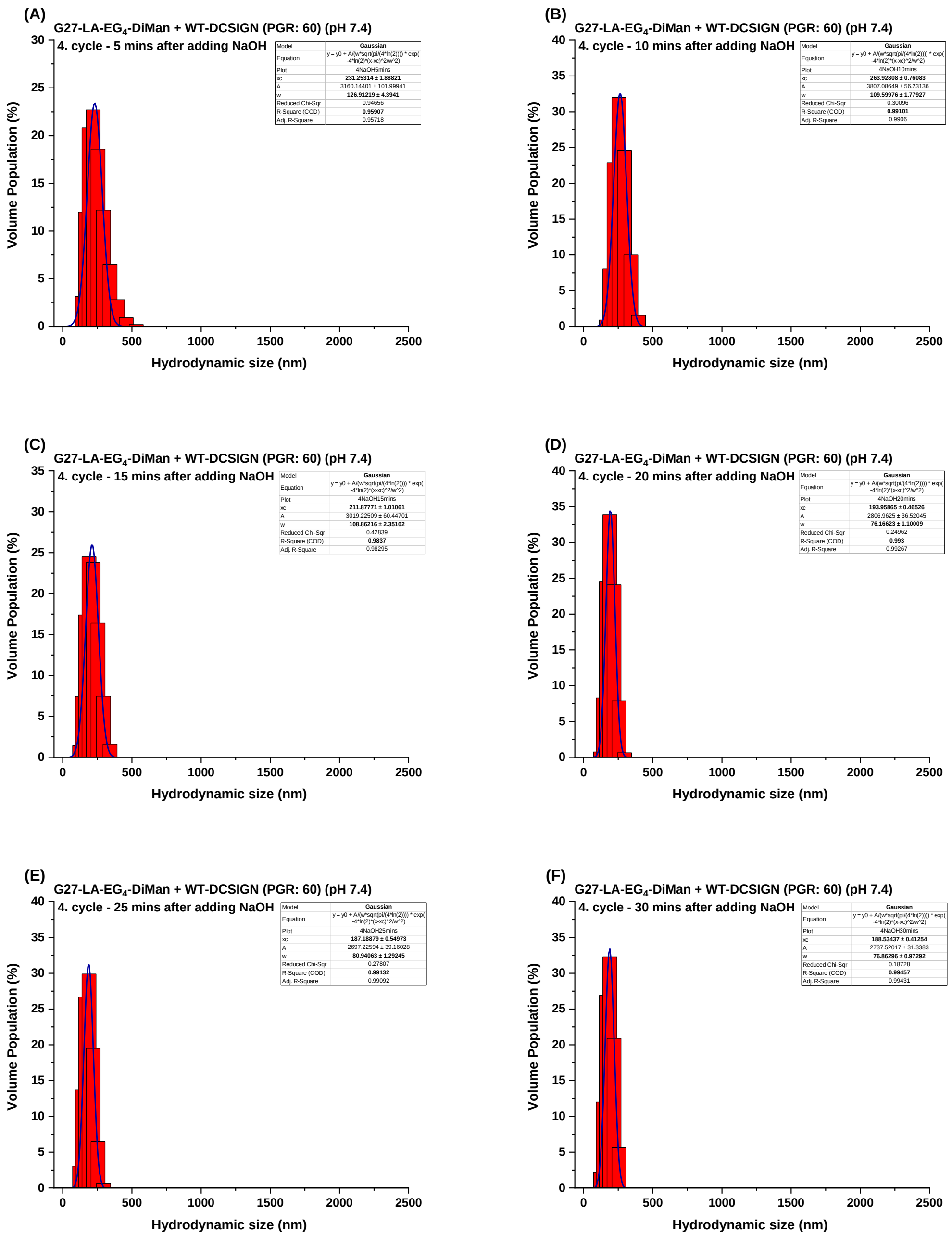

Figure S24. D_h histograms of G27-EG$_4$-DiMan (0.5 nM) + DC-SIGN (30 nM) sample as pH is switched from 5.0 to 7.4 by adding NaOH (cycle 4) over time: (A) 5 mins, (B) 10 mins; (C) 15 mins; (D) 20 mins; (E) 25 mins; (F) 30 mins.

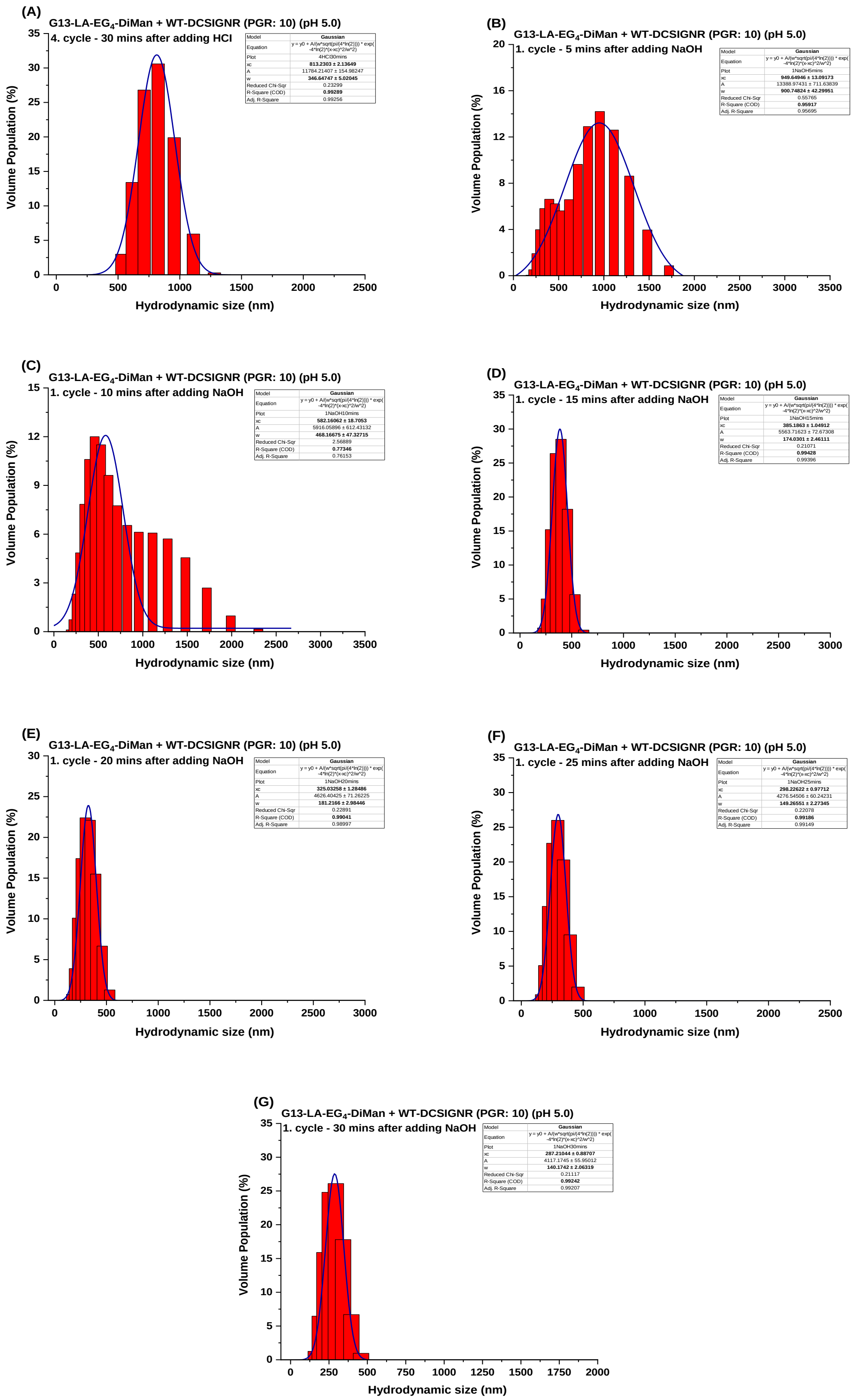

Figure S25. D_h histograms of G13-EG$_4$-DiMan (5 nM) +DC-SIGNR (50 nM) starting at pH 5.0 (A) after pH is switched to 7.4 by adding NaOH (cycle 1) over time: (B) 5 mins, (C) 10 mins; (D) 15 mins; (E) 20 mins; (F) 25 mins; (G) 30 mins.

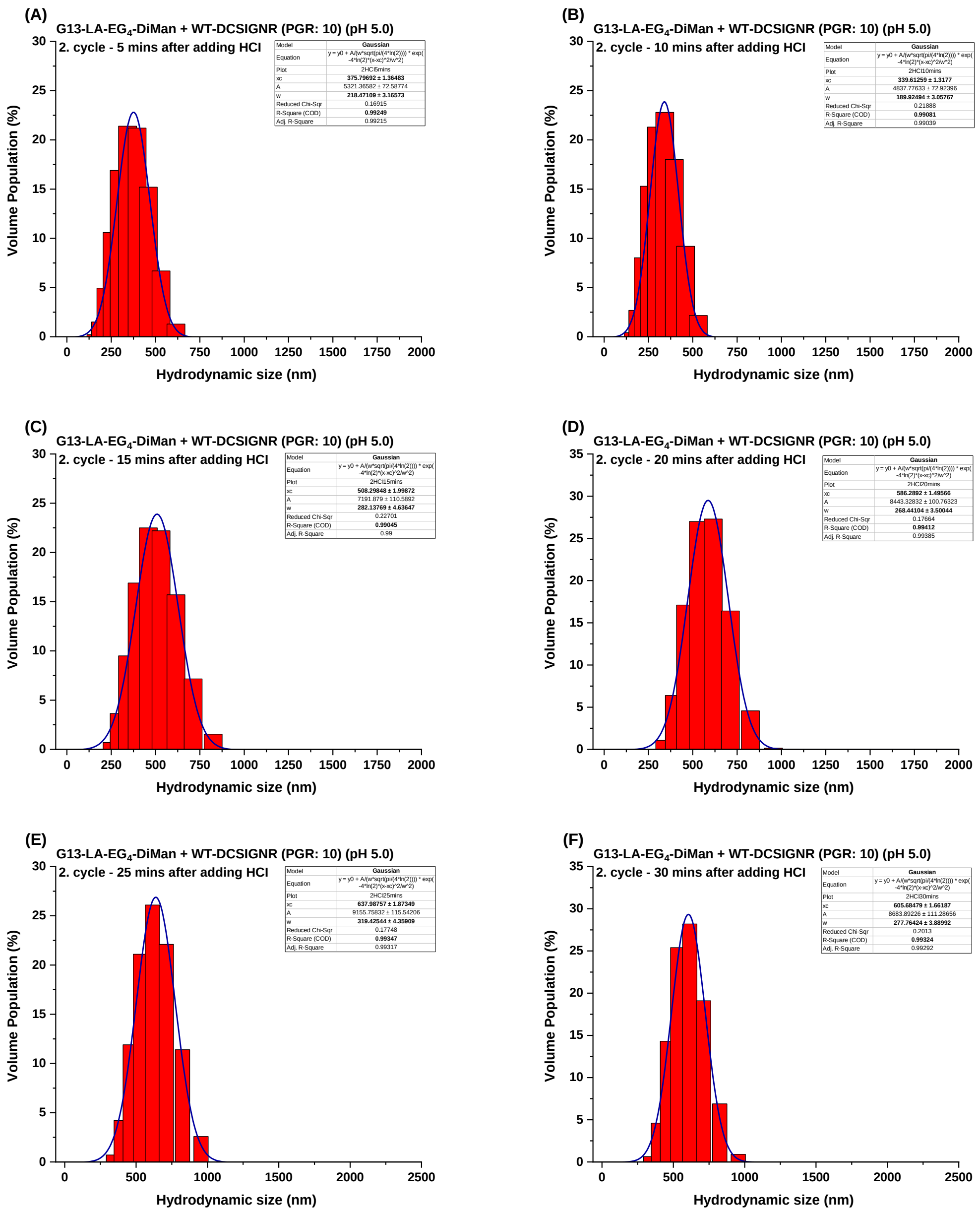

Figure S26. D_h histograms of G13-EG$_4$-DiMan (5 nM) +DC-SIGNR (50 nM) after pH is switched from 7.4 to 5.0 by adding HCl (cycle 2) over time: (A) 5 mins, (B) 10 mins; (C) 15 mins; (D) 20 mins; (E) 25 mins; (F) 30 mins.

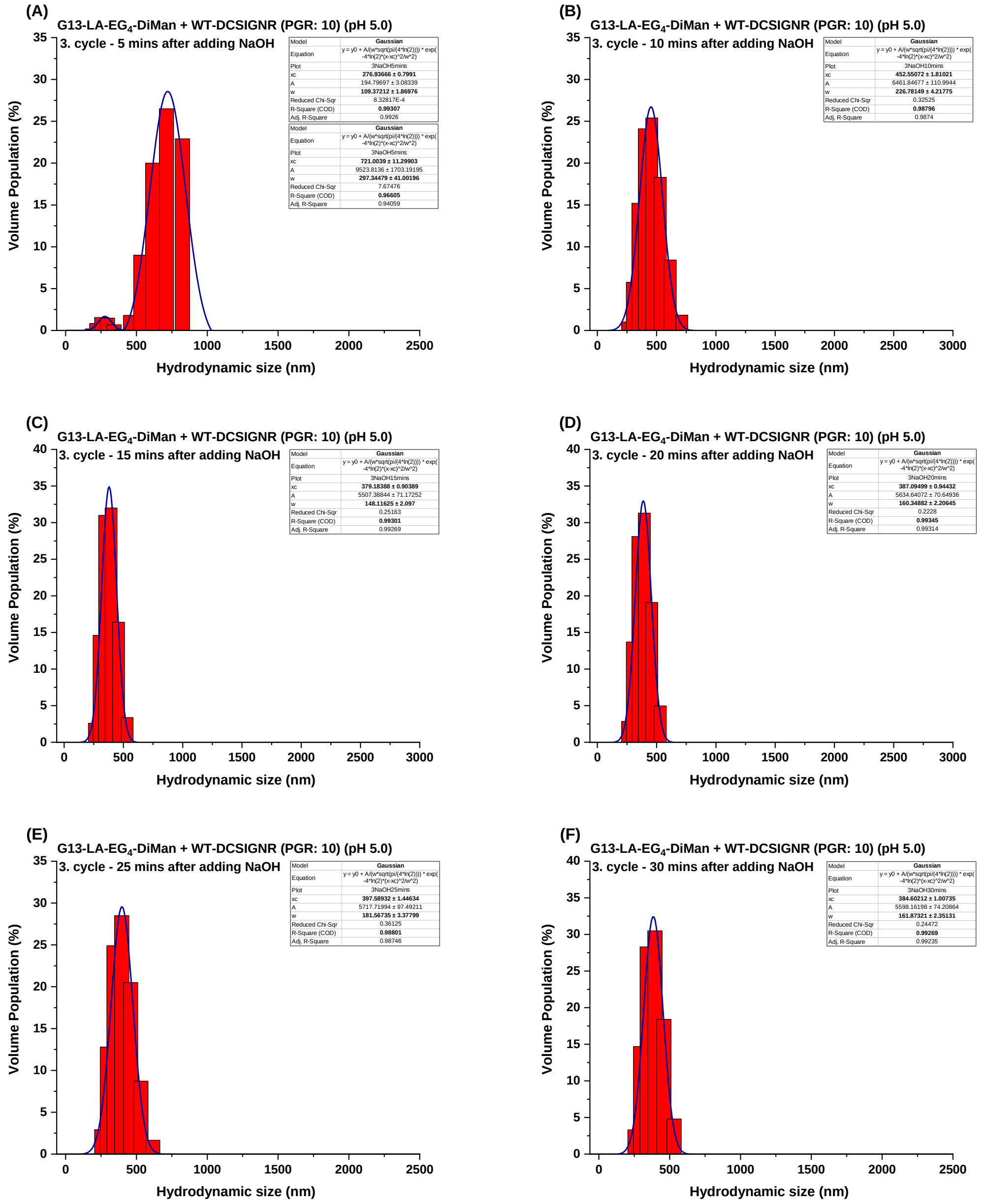

Figure S27. D_h histograms of G13-EG$_4$-DiMan (5 nM) +DC-SIGNR (50 nM) after pH is switched from 5.0 to 7.4 by adding NaOH (cycle 3) over time: (A) 5 mins, (B) 10 mins; (C) 15 mins; (D) 20 mins; (E) 25 mins; (F) 30 mins.

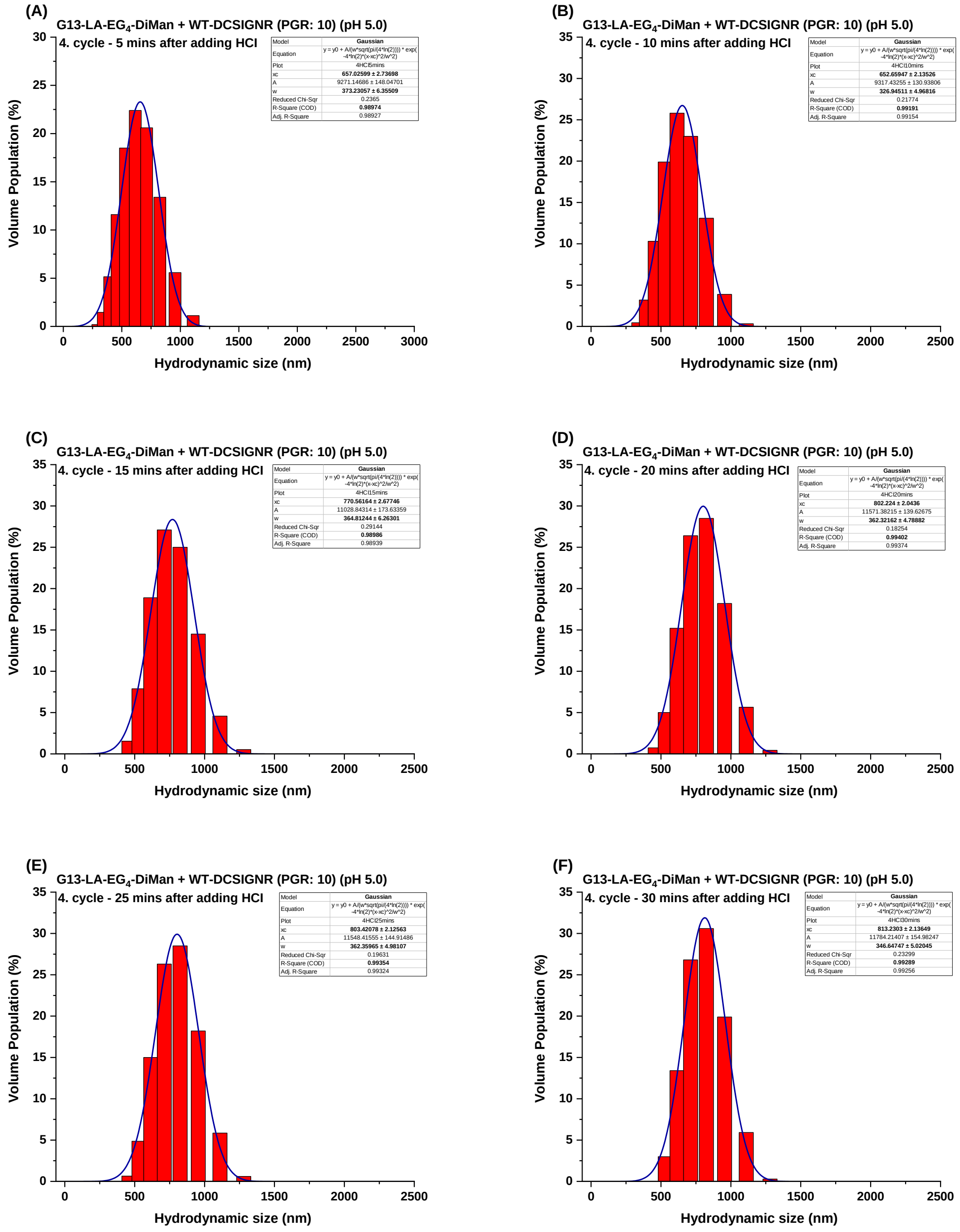

Figure S28. D_h histograms of G13-EG$_4$-DiMan (5 nM) +DC-SIGNR (50 nM) after pH is switched from 7.4 to 5.0 by adding HCl (cycle 4) over time: (A) 5 mins, (B) 10 mins; (C) 15 mins; (D) 20 mins; (E) 25 mins; (F) 30 mins.

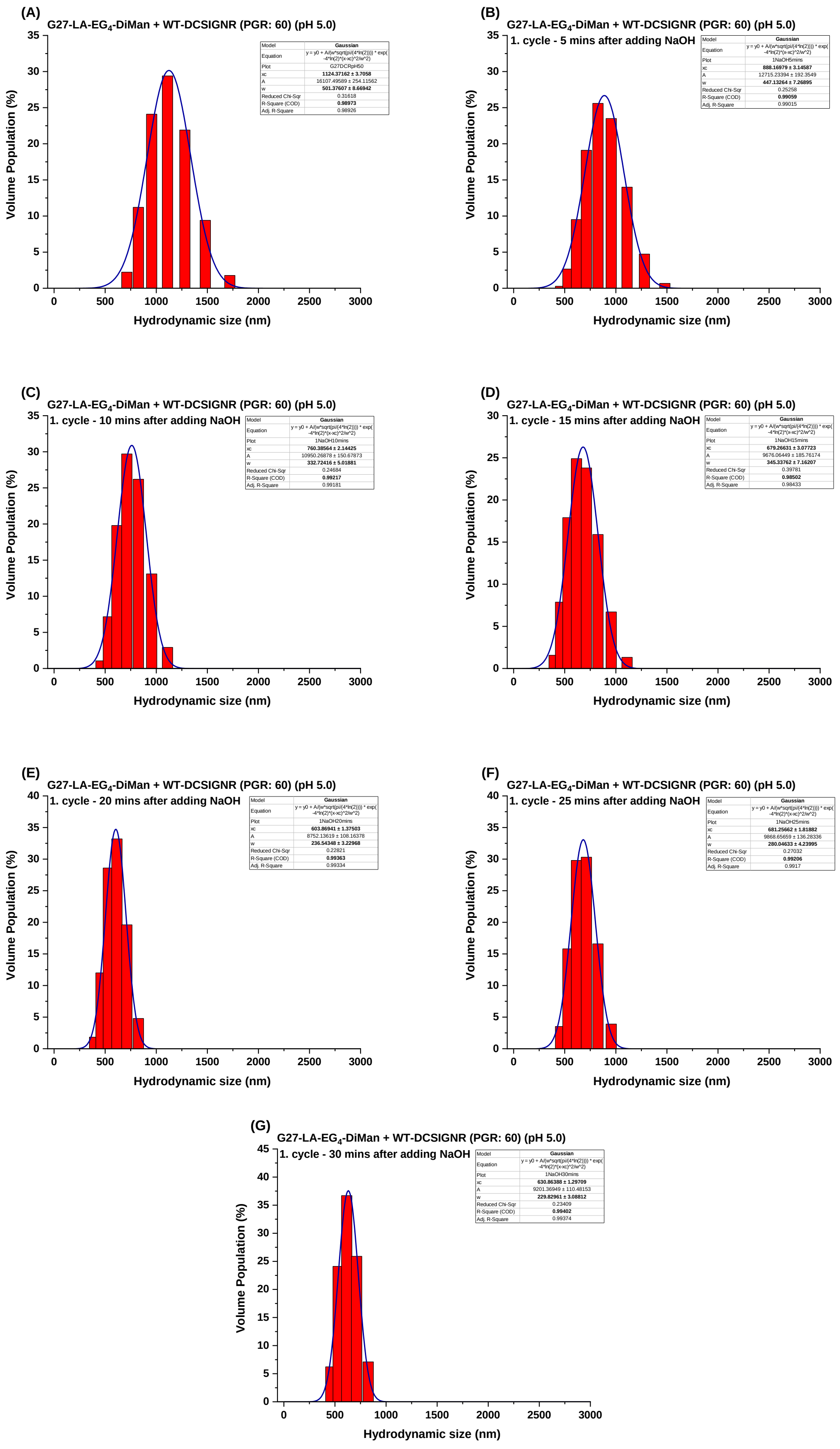

Figure S29. D_h histograms of G27-EG$_4$-DiMan (0.5 nM) + DC-SIGNR (30 nM) starting at pH 5.0 (A) after pH is switched to 7.4 by adding NaOH (cycle 1) over time: (B) 5 mins, (C) 10 mins; (D) 15 mins; (E) 20 mins; (F) 25 mins; (G) 30 mins.

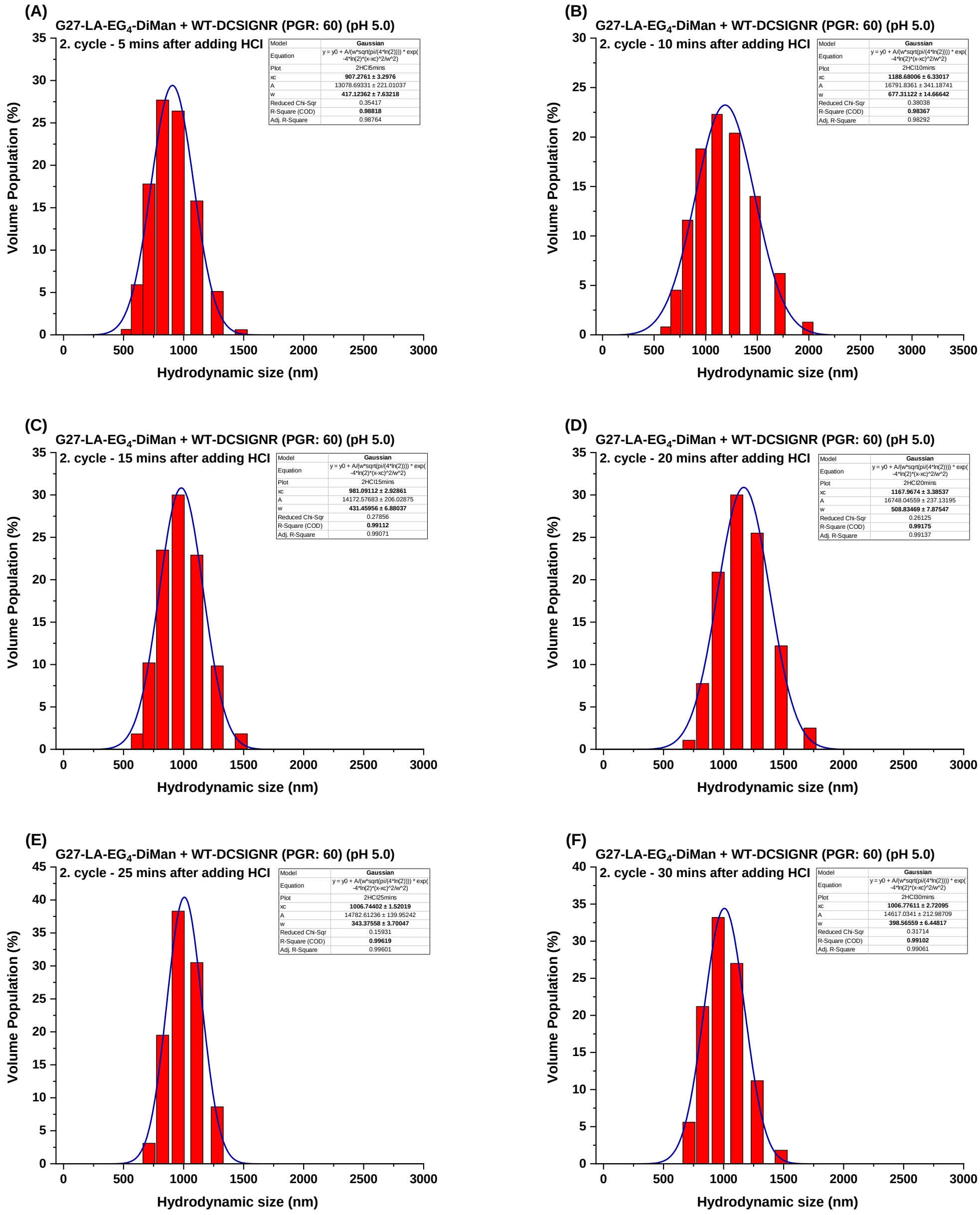

Figure S30. D_h histograms of G27-EG_4-DiMan (0.5 nM) +DC-SIGNR (30 nM) after pH is switched from 7.4 to 5.0 by adding HCl (cycle 2) over time: (A) 5 mins, (B) 10 mins; (C) 15 mins; (D) 20 mins; (E) 25 mins; (F) 30 mins.

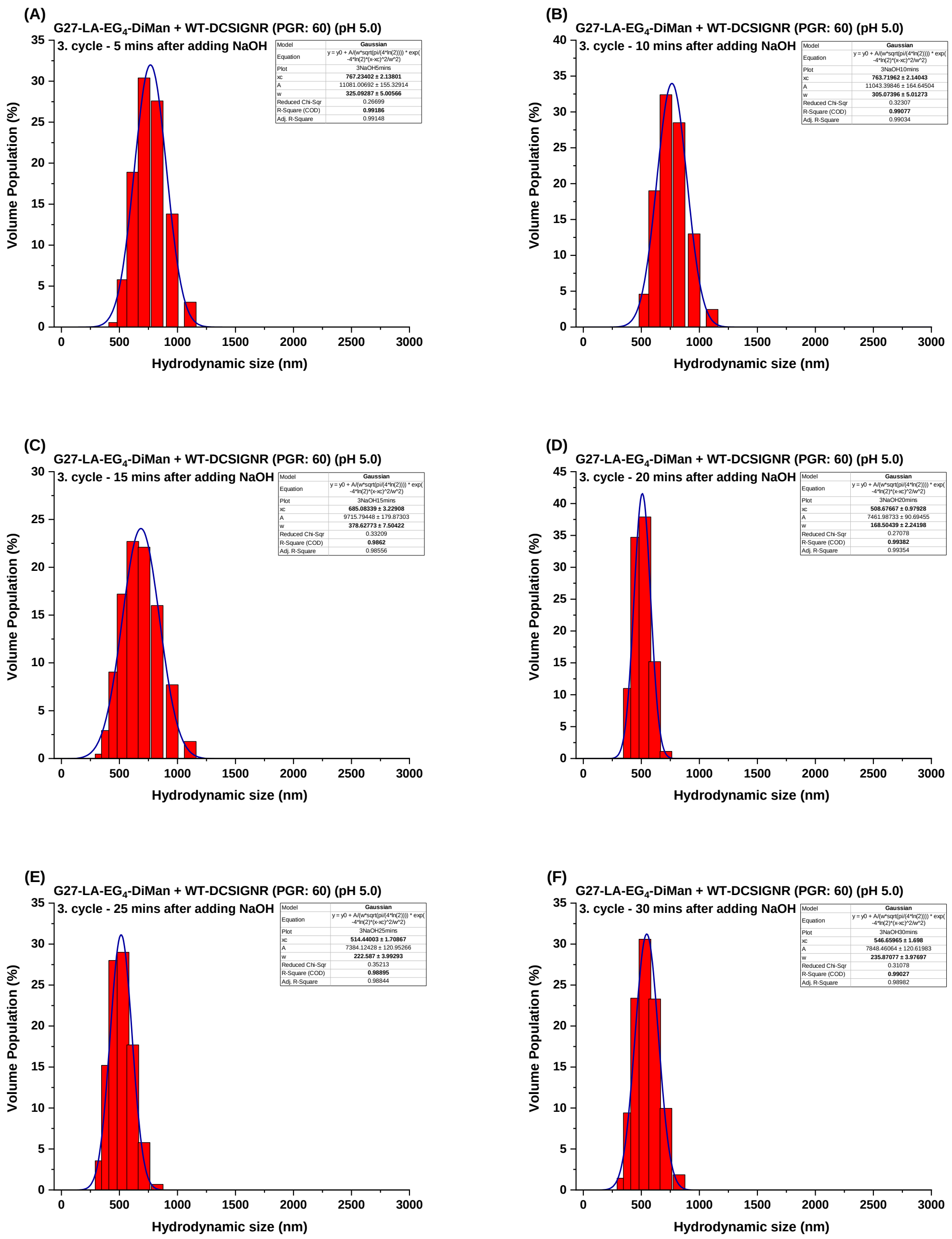

Figure S31. D_h histograms of G27-EG_4-DiMan (0.5 nM) +DC-SIGNR (30 nM) after pH is switched from 5.0 to 7.4 by adding NaOH (cycle 3) over time: (A) 5 mins, (B) 10 mins; (C) 15 mins; (D) 20 mins; (E) 25 mins; (F) 30 mins.

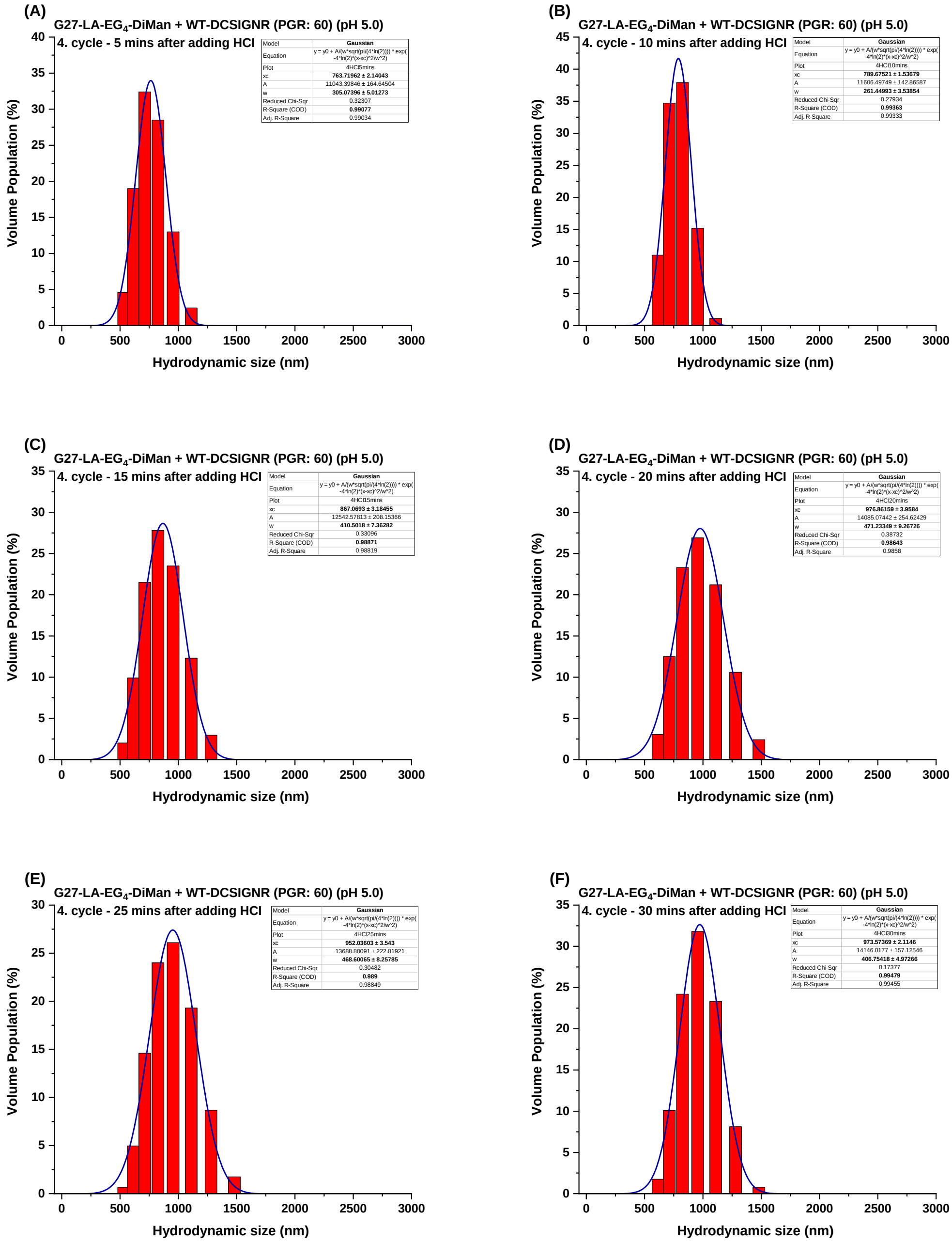

Figure S32. D_h histograms of G27-EG$_4$-DiMan (0.5 nM) +DC-SIGNR (30 nM) after pH is switched from 7.4 to 5.0 by adding HCl (cycle 4) over time: (A) 5 mins, (B) 10 mins; (C) 15 mins; (D) 20 mins; (E) 25 mins; (F) 30 mins.

10. Supporting References

1. X. Ji, X. Song, J. Li, Y. Bai, W. Yang and X. Peng, *J. Am. Chem. Soc.*, 2007, **129**, 13939-13948.
2. D. Budhadev, E. Poole, I. Nehlmeier, Y. Liu, J. Hooper, E. Kalverda, U. S. Akshath, N. Hondow, W. B. Turnbull and S. Pöhlmann, *J. Am. Chem. Soc.*, 2020, **142**, 18022-18034.
3. Y. Guo, I. Nehlmeier, E. Poole, C. Sakonsinsiri, N. Hondow, A. Brown, Q. Li, S. Li, J. Whitworth, Z. Li, A. Yu, R. Brydson, W. B. Turnbull, S. Pöhlmann and D. Zhou, *J. Am. Chem. Soc.*, 2017, **139**, 11833-11844.
4. L. Song, V. H. B. Ho, C. Chen, Z. Q. Yang, D. S. Liu, R. J. Chen and D. J. Zhou, *Adv. Healthc. Mater.*, 2013, **2**, 275-280.
5. Y. Guo, C. Sakonsinsiri, I. Nehlmeier, M. A. Fascione, H. Zhang, W. Wang, S. Pöhlmann, W. B. Turnbull and D. Zhou, *Angew. Chem. Int. Ed.*, 2016, **55**, 4738-4742.
6. H. D. Hill, J. E. Millstone, M. J. Banholzer and C. A. Mirkin, *ACS Nano*, 2009, **3**, 418-424.
